# Integrated transcriptomics, metabolomics and physiological analyses reveal differential response mechanisms of wheat to cadmium and/or salinity stress

**DOI:** 10.3389/fpls.2024.1378226

**Published:** 2024-10-01

**Authors:** Zonghao Yue, Yongchuang Liu, Limin Zheng, Qiaoyang Zhang, Yifan Wang, Yuwen Hao, Mengke Zhang, Yanjuan Chen, Zhengbing Wang, Le He, Keshi Ma

**Affiliations:** ^1^ College of Life Sciences and Agronomy, Zhoukou Normal University, Zhoukou, China; ^2^ School of Mechanical and Electrical Engineering, Zhoukou Normal University, Zhoukou, China

**Keywords:** *Triticum aestivum*, Cadmium, NaCl, multiple stresses, omics

## Abstract

Many soils face dual challenges of cadmium (Cd) contamination and salinization. However, the response of crops, especially wheat, to combined Cd and salinity stress is not understood. Here, wheat was grown in a hydroponic model for 14 days under single and combined Cd and NaCl stresses. Growth parameters, tissue Cd^2+^ and Na^+^ contents, and leaf chlorophyll (Chl), O2^•−^, and MDA levels were determined. Comparative transcriptomic and metabolomic analyses of the leaves were performed. The results showed that combined stress had a greater inhibitory effect on Chl contents and generated more O2^•−^ and MDA, resulting in more severe wheat growth retardation than those under Cd or NaCl stress. Stress-induced decrease in Chl levels may be attributed to the inhibition of Chl biosynthesis, activation of Chl degradation, or a decline in glutamate content. Cd addition weakened the promotional effect of NaCl on SOS1 gene expression, thereby increasing the Na^+^ content. Contrastingly, NaCl supplementation downregulated the Nramp and ZIP gene expressions related to Cd uptake and transport, thereby impeding Cd^2+^ accumulation. All stresses enhanced tryptophan content via promoting tryptophan biosynthesis. Meanwhile, Cd and NaCl stresses activated phenylpropanoid biosynthesis and purine metabolism, respectively, thereby increasing the levels of caffeic acid, fumaric acid, and uric acid. Activating the TCA cycle was important in the wheat’s response to combined stress. Additionally, NaCl and combined stresses affected starch and sucrose metabolism, resulting in sucrose and trehalose accumulation. Our findings provide a comprehensive understanding of the response of wheat to the combined Cd and salinity stress.

## 1 Introduction

Cadmium (Cd) is a non-essential heavy metal in organisms. Owing to mining and smelting activities, electroplating, industrial solid waste discharge, sewage irrigation, and overuse of pesticides and fertilizers, large amounts of Cd are released into the environment, especially into the soil (Uchimiya et al., 2020). According to a national soil pollution survey report announced by China’s Ministry of Environmental Protection, soil Cd has the highest over-standard rate (7.0%) among heavy metals contamination. Cd concentrations in soils (including agricultural soil) of the area around a Pb-zinc minein Liuzhou, China and a lead-zinc smelter in Zhuzhou, China ranged from 6.56–17.93 and 0.11–23.3 mg·kg^-1^, respectively (Yu et al., 2021; Zhou et al., 2022). In addition to China, other countries worldwide such as Japan, Thailand, Korea, Pakistan, India, Vietnam, and Peru also face soil Cd contamination (Arao et al., 2010; Sriprachote et al., 2012; Ahn et al., 2017; Rehman et al., 2017; Kumar et al., 2019; Nguyen et al., 2020; Thomas et al., 2023). As a highly toxic and non-biodegradable heavy metal, Cd damages crop growth and easily accumulates in crops, thereby endangering human health through the food chain (Hu et al., 2022). Therefore, Cd contamination has attracted considerable attention worldwide.

In addition to Cd contamination, soil salinization is a non-negligible abiotic stress for crops. Saline soils are estimated to cover 833 million hectares worldwide (https://www.fao.org). Even worse, saline agricultural soils are increasing at a rate of 10% every year due to climate change, brackish water irrigation, and industrial pollution (Li et al., 2021). High salinity often results in crop growth inhibition and yield loss via ionic toxicity, osmotic stress, and oxidative stress, causing economic losses exceeding 27.3 billion dollars annually (Qadir et al., 2014; Yang and Guo, 2018; Zhu, 2016).

Increasing evidences revealed that many soils were suffering from the dual challenges of Cd contamination and salinity stress, particularly in arid and semi-arid regions and coastal areas (Cheng et al., 2019; Lutts and Lefèvre, 2015). For instance, high levels of Cd (4.17 mg·kg^-1^) and salinity (EC 4.67 dS·m^-1^) were detected simultaneously in the soil from Murcia and its surroundings in Spain (Acosta et al., 2011). Furthermore, high soil Cd (2.0 mg·kg^-1^) and salinity (EC 6.55 dS·m^-1^) were also observed around an abandoned factory in Northern China (Wu et al., 2022). Therefore, crops may be subjected to dual stress from Cd and salinity.

Wheat (*Triticum aestivum* L.) is the most cultivated edible cereal globally, providing 20% of the daily calories and protein requirements for humans (Tricker et al., 2018). In 2021, the total wheat cultivation area was 220.8 million hectares, producing 770.9 million tons (https://www.fao.org/faostat). Since wheat is a moderately salinity-tolerant crop with strong Cd enrichment ability (Li and Zhou, 2019; Quan et al., 2021), it is of great significance to study the response of wheat to combined Cd and salinity stress to ensure food security and cultivate Cd- and salinity-tolerant varieties. However, studies on Cd and salinity combination in wheat are limited, mainly focusing on antioxidant properties (Abbas et al., 2018; Mühling and Läuchli, 2003; Shafi et al., 2009). Little is known about the transcriptomic and metabolomic mechanisms underlying the wheat response to a combination of Cd and salinity stresses.

In this study, Zhoumai 36, a winter wheat cultivar planted in the Huang-Huai-Hai region, China, was exposed to single and combined stresses of Cd and salinity for 14 days using a hydroponic model. The growth parameters were recorded, and leaf chlorophyll (Chl), superoxide anion (O2^•−^), malondialdehyde (MDA), tissue Na^+^, and tissue Cd^2+^ contents were measured. Comparative transcriptomic and metabolomic analyses of the leaves were conducted to reveal the underlying response mechanisms of wheat to single and combined stresses. The findings of this study will improve our understanding of wheat response and adaptation to combined Cd and salinity stress.

## 2 Materials and methods

### 2.1 Plant culture and exposure experiment

Wheat cultivar Zhoumai 36 seeds were surface-sterilized and germinated, as described in our previous study (Yue et al., 2022). Ten well-germinated seeds were transferred to each glass tissue culture bottle containing 325 mL of 1/2 Hoagland solution. The bottles were randomly assigned to four groups, all in quintuplicates: (1) Control group: no addition of NaCl and Cd^2+^; (2) NaCl group: 150 mM NaCl; (3) Cd group: 100 μM Cd^2+^; and (4) Combined NaCl+Cd group: 150 mM NaCl and 100 μM Cd^2+^. To avoid osmotic shock, NaCl (2.925 g) is added into the nutrient solution in increments of 50 mM every 24 h to reach the final concentration of 150 mM. Cd^2+^ was added as CdCl_2_·2.5 H_2_O. The NaCl concentration was adopted from our previous study (Yue et al., 2022), and the Cd concentration was selected based on the Cd tolerance experiment of Zhoumai 36 (**Supplementary Figure S1**) and soil Cd content in China (Yu et al., 2021; Zhou et al., 2022). The hydroponics experiment was performed for 14 days under 25 ± 0.5°C, 60 ± 3% relative humidity, and 14/10 h of light/dark period. The nutrient solution was supplemented daily to compensate for water loss caused by evaporation and plant transpiration and to ensure plants received as consistent nutrition and water as possible throughout the experiment.

### 2.2 Sample collection and growth parameters measurement

After 14 days of treatment, the wheat seedlings were harvested and washed thrice with deionized water. The primary root length and shoot height were measured and recorded. Subsequently, the roots and leaves were separated. A portion of the roots and leaves were dried for 48 h at 75°C in an oven and used to determine the elemental content after weighing the dry weight. The remaining leaves were immediately frozen in liquid nitrogen and stored at -80°C until biochemical, transcriptomic and metabolomic analyses.

### 2.3 Measurement of Chl content

Chl was extracted as previously described (Hiscox and Israelstam, 1979). Detailed procedures can be found in the **Supplementary Methods**. The absorbance of the extract solutions was measured at 645 and 663 nm using a SpectraMax i3x microplate reader (Molecular Devices, San Jose, USA). Finally, the total Chl, Chl *a*, and Chl *b* contents were calculated according to established equations (Arnon, 1949).

### 2.4 Measurement of tissues Na^+^ and Cd^2+^ content

Oven-dried root and shoot samples were digested in glass digestion tubes containing 8 mL concentrated HNO_3_ and 2 mL H_2_O_2_. The digestion was performed in a SPH108 digestion instrument (ALVA Instrument Co., Ltd., Jinan, China) under the following conditions: 12 min at 130°C, and 50–60 min at 180°C. Subsequently, the digested samples were diluted to 45 mL with deionized water and filtered using a 0.45 μm filter membrane. The Na^+^ and Cd^2+^ contents were determined using an A3AFG flame atomic absorption spectrophotometer (Purkinje General Instrument Co., Ltd., Beijing, China).

### 2.5 Determination of O^2•−^ and MDA contents

O^2•−^ content was determined using a superoxide anion content assay kit (Boxbio, Beijing, China). MDA content was determined using an MDA colorimetric assay kit (Elabscience, Wuhan, China). Briefly, 0.1 g fresh leaf tissue was homogenized with 1 mL PBS buffer (0.01M, pH7.4) and centrifuged for 10,000× *g* at 4°C for 20 min. The resulting supernatant was collected, and the O_2_^•−^ and MDA contents were measured according to the manufacturer’s instructions. The supernatant protein concentration was determined by using a BCA protein colorimetric assay kit (Elabscience).

### 2.6 Leaf transcriptome sequencing, bioinformatics analysis, and quantitative real-time PCR validation

Total RNA was extracted from the leaf samples (n = 3 per group), and RNA quality assessment was performed as previously described (Yue et al., 2022). High-quality total RNA (1 μg) was used to construct RNA-seq transcriptome libraries using Stranded mRNA Prep Ligation (Illumina, San Diego, USA). Detailed library construction procedures are provided in the **Supplementary Methods**. After quantification by Qubit 4.0 Fluorometer (Thermo Fisher, Waltham, USA), the paired-end libraries were sequenced in an Illumina Novaseq 6000 system by Majorbio Biotech Co., Ltd, Shanghai, China.

Raw reads were processed using fastp software to remove the adapter, low-quality, higher N-ratio (> 10%), and <20 bp reads. The resulting clean reads were aligned with the wheat reference genome (version IWGSC) using HISAT2 software and assembled using StringTie software. Gene expression levels were calculated using the transcript per million reads method in RSEM software. Differentially expressed genes (DEGs) between different treatment groups were identified using DESeq2 software, and the screening criteria were *p* adjust < 0.01 and |Log_2_ (fold change [FC])| ≥ 1.0. Gene ontology (GO) and Kyoto Encyclopedia of Genes and Genomes (KEGG) pathway enrichment analyses of these DEGs were conducted at *p* adjust < 0.05 using Goatools and KOBAS software, respectively. Besides, the protein-protein interaction (PPI) network of DEGs in the key pathways was analyzed based on the STRING database and visualized using the NetworkX package. The raw data were uploaded to the NCBI Sequence Read Archive database under the project accession number PRJNA1020113.

Six DEGs (3 up-regulated and 3 down-regulated) were randomly selected to verify the accuracy of the transcriptome data using qPCR, as previously described (Yue et al., 2021). All primer sequences are listed in **Supplementary Table S1**.

### 2.7 Leaf non-targeted metabolomics and data analysis

Briefly, 50 mg frozen leaf samples (n = 6 per group) were homogenized with 400 µL 80% methanol (containing 0.02 mg·mL^-1^ L-2-chlorophenylalanine) for 6 min at -10°C in a Wonbio-96c tissue grinder (Wonbio, Shanghai, China). The homogenized solutions were ultrasonicated for 30 min at 5°C by an SBL-10DT ultrasonic cleaner (Scientz, Ningbo, China). They were then maintained at -20°C for 30 min and centrifuged at 13,000× *g* for 15 min at 4°C. Finally, the supernatant was transferred to an injection vial for ultra-high performance liquid chromatography tandem mass spectrometry (UPLC-MS/MS) analysis. Detailed UPLC-MS/MS procedures are provided in the **Supplementary Methods** and **Supplementary Tables S2**, **S3**.

The raw UPLC-MS/MS data were pretreated using Progenesis QI 2.3 software (Waters, Milford, USA), and a data matrix containing the retention time, mass-to-charge (m/z) ratio, and peak intensity was generated. Subsequently, the data matrix was uploaded to the Majorbio Cloud platform (https://cloud.majorbio.com) for data analysis. Principal component analysis (PCA) and orthogonal projections to latent structure-discriminant analysis (OPLS-DA) were performed using the ropls (Version 1.6.2) R package. Differentially abundant metabolites (DAMs) between different treatments were identified based on variable importance in the projection (VIP) > 1 (obtained by the OPLS-DA model) and *p* < 0.05 (generated by Student’s test). Pathway enrichment analysis of DAMs was conducted using the KEGG database. The raw data were uploaded to the EMBL-EBI MetaboLights database with the accession number MTBLS9697.

### 2.8 Statistical analysis

Data are expressed as means ± standard deviation (SD). Significant differences between treatment groups were assessed by one-way analysis of variance (ANOVA) with Tukey’s multiple comparisons test using SPSS statistics software 19.0 (IBM, Armonk, USA). In addition, Pearson’s correlation analysis was also performed using SPSS 19.0. *p* < 0.05 and *p* < 0.01 were considered statistically significant and extremely significant, respectively.

## 3 Results

### 3.1 Effect of Cd, NaCl, and combined stresses on wheat seedling growth parameters

**Figure 1:**
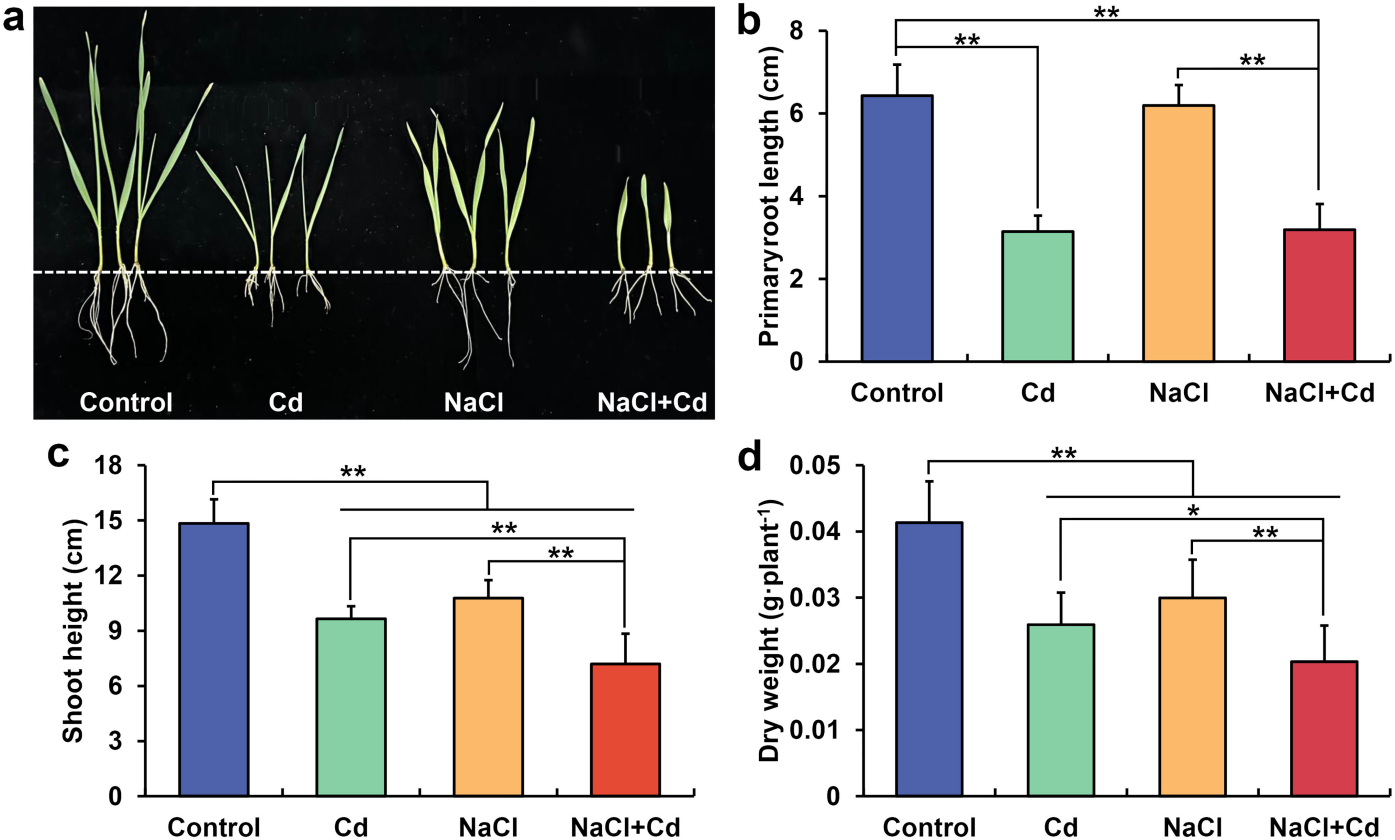
Adverse effects of Cd, NaCl, and NaCl+Cd stresses on wheat seedling growth. **(A)** Photograph of wheat seedlings; **(B)** Primary root length of wheat plants; **(C)** Shoot height of wheat plants; **(D)** Dry weights of wheat plants. Data are expressed as the means ± SD (n = 15 per group). * and ** represent *p* < 0.05 and *p* < 0.01, respectively.

The growth of wheat seedlings was significantly inhibited under Cd, NaCl, and combined stresses compared with that in the control group (**Figure 1A**). Specifically, primary root length (PRL) significantly decreased in the presence of Cd and NaCl+Cd (**Figure 1B**). Shoot height (SH) and plant dry weight (PDW) significantly decreased under all three stress treatments (**Figures 1C, D**). Notably, combined stress showed more severe inhibition of PRL (except for Cd *vs*. NaCl+Cd), SH, and PDW than Cd or NaCl stress (**Figures 1B-D**).

### 3.2 Leaf transcriptomic analysis under Cd, NaCl, and combined stresses

**Figure 2:**
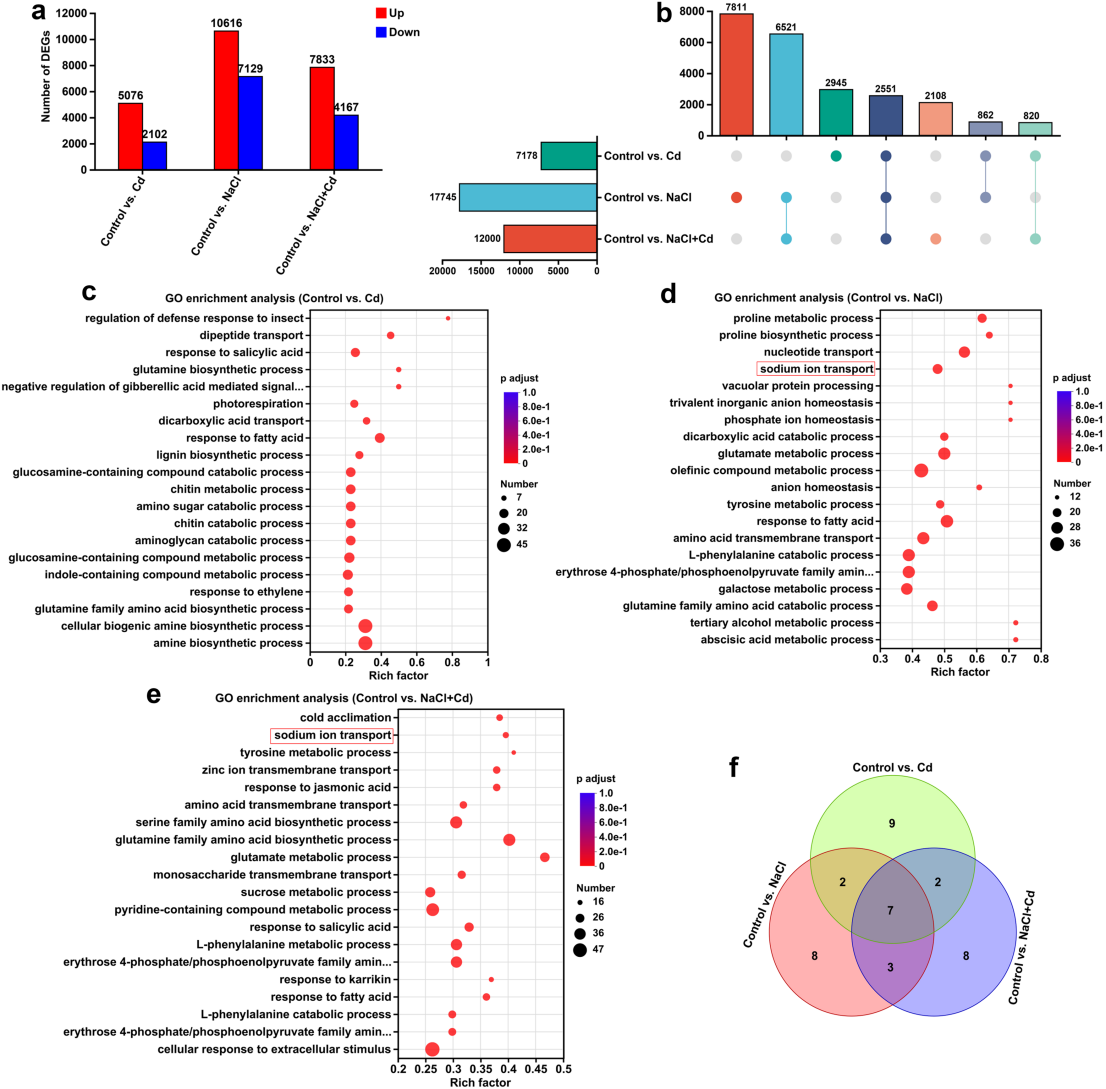
Transcriptomic analysis of wheat leaf under Cd, NaCl, and NaCl+Cd stresses. **(A)** Statistical graph of DEGs under Cd, NaCl, and combined stresses compared to those in the Control group. Red bars represent upregulated genes, and blue bars represent downregulated genes. **(B)** UpSet plot of DEGs. **(C-E)** GO enrichment analysis of DEGs in response to Cd **(C)**, NaCl **(D)**, and combined **(E)** stresses. The size of the dots represents the number of DEGs enriched in the GO term, and *p* adjust < 0.05 represents statistical significance. **(F)** Venn diagram of the top 20 significantly enriched KEGG pathways among the three stress treatments.

To analyze the responses of global gene expression in leaves to Cd, NaCl, and combined stresses, RNA-seq was performed; an overview of the RNA-seq results is presented in **Supplementary Tables S4**-**S6**. In total, 7,178 (5,076 upregulated and 2,102 downregulated), 17,745 (10,616 upregulated and 7,129 downregulated), and 12,000 (7,833 upregulated and 4,167 downregulated) DEGs were identified in the control *vs*. Cd, control *vs*. NaCl, and control *vs*. NaCl+Cd comparison groups, respectively (**Figure 2A**; **Supplementary Figure S2**). Among these DEGs, 2,551 genes (10.80%) were common to all stress groups, whereas 2,945 (12.47%), 7,811 (33.07%), and 2,108 (8.93%) genes were unique to the Cd, NaCl, and combined stress groups, respectively (**Figure 2B**). qPCR analysis showed similar results to those obtained from the RNA sequencing, indicating that the transcriptome data is reliable (**Supplementary Figure S3**).

GO enrichment analysis revealed that these DEGs in response to Cd, NaCl, and combined stresses were significantly enriched in 842, 1,371, and 1,110 GO terms, respectively (**Supplementary Tables S7**, **S8**). The top 20 GO terms are shown in **Figures 2C-E**. Notably, the GO terms related to Na^+^ transport, including “sodium ion transport”, “sodium:proton antiporter activity”, “sodium ion transmembrane transporter activity”, “sodium ion import across plasma membrane”, and “sodium ion transmembrane transport” were significantly enriched under NaCl and combined stresses. In addition, the GO terms “response to metal ion”, “metal ion transport”, “metal ion binding”, and “metal ion transmembrane transporter activity” were significantly enriched under three stresses. KEGG enrichment analysis revealed that the DEGs in response to Cd, NaCl, and combined stresses were significantly enriched in 44, 45, and 46 pathways, respectively (**Supplementary Tables S9**, **S10**). Among the top 20 pathways, 7 were common to all three stresses, while 9, 8, and 8 were unique to Cd, NaCl, and combined stresses, respectively (**Figure 2F**; **Supplementary Figure S4**). A detailed description is provided in the **Supplementary Results**.

### 3.3 Effect of Cd, NaCl, and combined stresses on Na^+^ and Cd^2+^ uptake in wheat seedlings

**Figure 3:**
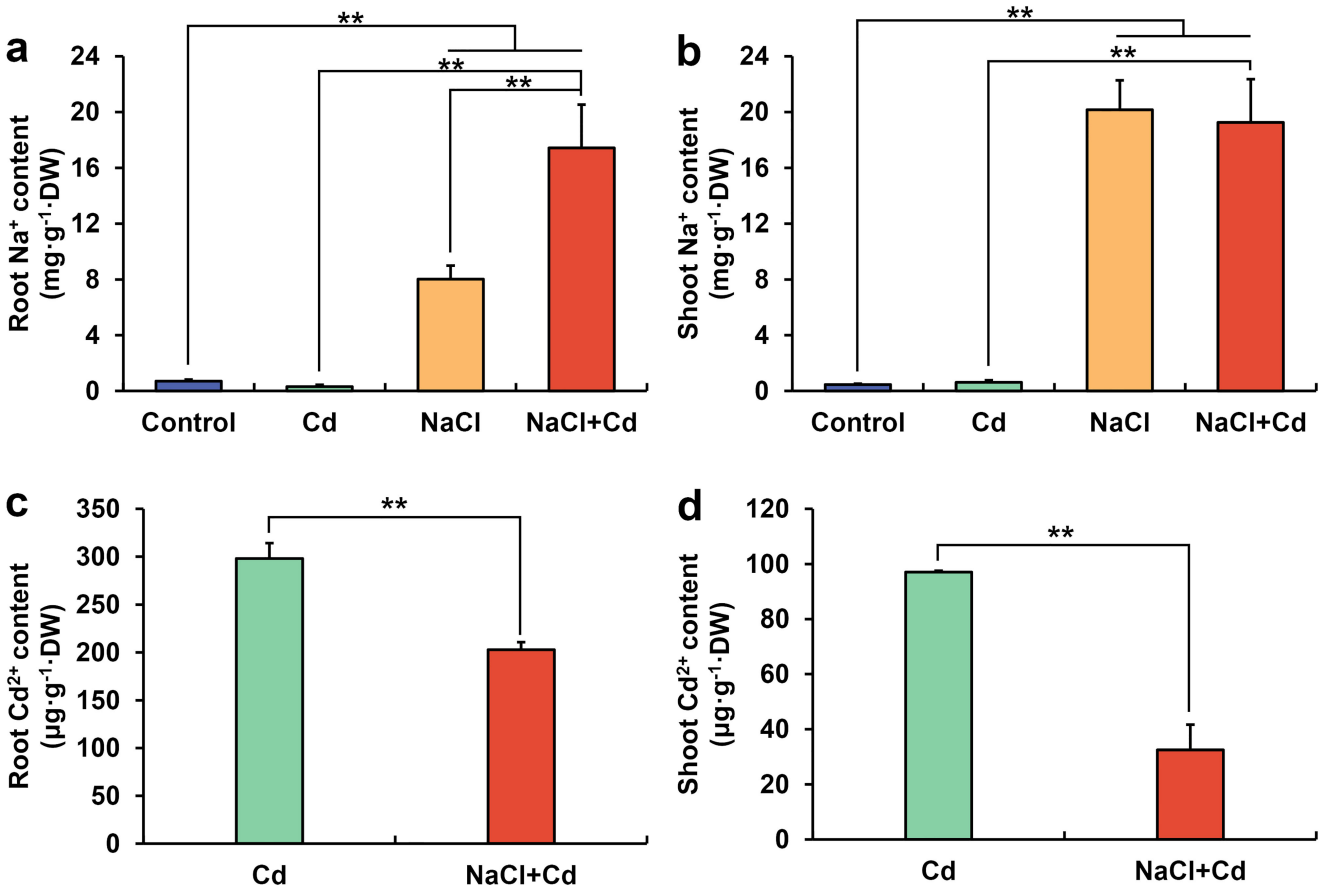
Root Na^+^ **(A)**, shoot Na^+^ **(B)**, root Cd^2+^ **(C)**, and shoot Cd^2+^ **(D)** contents in wheat seedlings under Cd, NaCl, and NaCl+Cd stresses. Data are expressed as the means ± SD (n = 3 per group). ** represent *p* < 0.01.

The Na^+^ content in the roots and shoots significantly increased under NaCl and combined stresses compared with those in the control group (**Figures 3A, B**). Notably, Na^+^ content in the roots under combined stress was 2.17-fold higher than that under NaCl stress. Cd^2+^ was not detected in the control and NaCl groups. The Cd^2+^ content in the roots and shoots significantly decreased under combined stress compared with that under Cd stress (**Figures 3C, D**).

A total of 25 and 20 DEGs were significantly enriched in GO terms related to Na^+^ transport under NaCl and combined stresses, respectively. Among these DEGs, some encoding salt overly sensitive 1 (SOS1), SOS2, and SOS3 were significantly upregulated under both stress treatments (**Table 1**). Remarkably, the relative expression level of these upregulated SOS1 genes under combined stress was only 0.74-fold that of NaCl stress alone. In addition, 7 and 3 DEGs encoding Na^+^/H^+^ antiporters (NHXs) were significantly upregulated under NaCl and combined stresses, respectively.

**Table 1:** DEGs related to Na^+^ transport under NaCl stress and combined stress.

Gene ID	Gene description	log2FC (NaCl/Control)	Regulation	log2FC (NaCl+Cd/Control)	Regulation
Salt overly sensitive (SOS) pathway					
TraesCS3D02G022900	SOS1	1.98	up	1.53	up
TraesCS7B02G475500	SOS1-like	1.22	up	1.13	up
TraesCS3B02G021600	SOS1-like			1.23	up
TraesCS7D02G374400	SOS2-like	1.29	up	1.42	up
TraesCS1D02G358400	SOS3-like			1.32	up
TraesCS1B02G370900	SOS3-like	3.04	up	2.61	up
Na+/H+ antiporters (NHXs)					
TraesCS4A02G145300	vacuolar NHX-like	1.60	up		
TraesCS4D02G147600	vacuolar NHX-like	2.03	up		
TraesCS2B02G141900	vacuolar NHX-like	2.83	up		
TraesCS2D02G123000	vacuolar NHX-like	1.80	up		
TraesCS4B02G125700	vacuolar NHX-like	1.24	up		
TraesCS2A02G121000	vacuolar NHX-like	3.32	up	3.83	up
TraesCS7B02G191300	vacuolar NHX-like	4.56	up	7.99	up
TraesCS7D02G226200	vacuolar NHX-like			6.18	up

Cd and combined stresses affected many DEGs related to Cd uptake and transport (**Table 2**). Under Cd stress, 5 DEGs encoding natural resistance-associated macrophage protein (Nramp) and 8 DEGs encoding zinc transporter (ZIP) were significantly upregulated. Under combined stress, 4 DEGs encoding Nramp (2 upregulated and 2 downregulated) and 20 DEGs (7 upregulated and 13 downregulated) encoding ZIP were significantly altered.

**Table 2:** DEGs related to Cd uptake and transport under Cd stress and combined stress.

Gene ID	Gene description	log2FC (Cd/Control)	Regulation	log2FC (NaCl+Cd/Control)	Regulation
Natural resistance-associated macrophage protein (Nramp)					
TraesCS7A02G327300	Nramp2	6.54	up		
TraesCS7B02G227900	Nramp2	10.33	up		
TraesCS7D02G451900	Nramp2	1.28	up		
TraesCS4A02G050500	Nramp2	2.28	up	3.06	up
TraesCS4D02G254100	Nramp2	2.48	up	1.75	up
TraesCS3A02G195100	Nramp2			-2.66	down
TraesCS3D02G206000	Nramp2			-2.80	down
Zinc/iron-regulated transporter-like protein (ZIP)					
TraesCS1D02G294000	ZIP, 1/2/3	3.36	up		
TraesCS4A02G294300	ZIP_1_2_3	6.79	up		
TraesCS7B02G266800	ZIP_1_2_3	1.42	up		
TraesCS7B02G321200	ZIP_1_2_3	2.34	up		
TraesCS3A02G527000	ZIP_1_2_3	8.38	up	6.83	up
TraesCS3B02G595100	ZIP_1_2_3	7.63	up	5.95	up
TraesCS3D02G532400	ZIP_1_2_3	6.78	up	5.44	up
TraesCS7D02G362100	ZIP_1_2_3	1.23	up	-1.33	down
TraesCS1A02G125500	ZIP_1_2_3			-2.05	down
TraesCS1A02G297500	ZIP_1_2_3			-2.98	down
TraesCS1B02G144500	ZIP_1_2_3			-2.26	down
TraesCS1B02G306500	ZIP_1_2_3			-2.62	down
TraesCS1D02G128000	ZIP_1_2_3			-1.84	down
TraesCS1D02G293900	ZIP_1_2_3			-2.19	down
TraesCS2B02G533800	ZIP_1_2_3			-3.82	down
TraesCS2D02G506300	ZIP_1_2_3			-2.10	down
TraesCS7A02G361000	ZIP_1_2_3			-1.66	down
TraesCS7A02G420200	ZIP_1_2_3			-2.54	down
TraesCS2A02G424200	ZIP_1_2_3			-3.31	down
TraesCS2D02G422000	ZIP_1_2_3			-2.00	down
TraesCS7A02G340000	ZIP2			2.72	up
TraesCS1D02G112500	ZIP6-like			1.43	up
TraesCS1A02G111000	ZIP6-like			1.04	up
TraesCS1B02G128800	ZIP6-like			1.75	up

### 3.4 Leaf metabolic analysis under Cd, NaCl, and combined stresses

**Figure 4:**
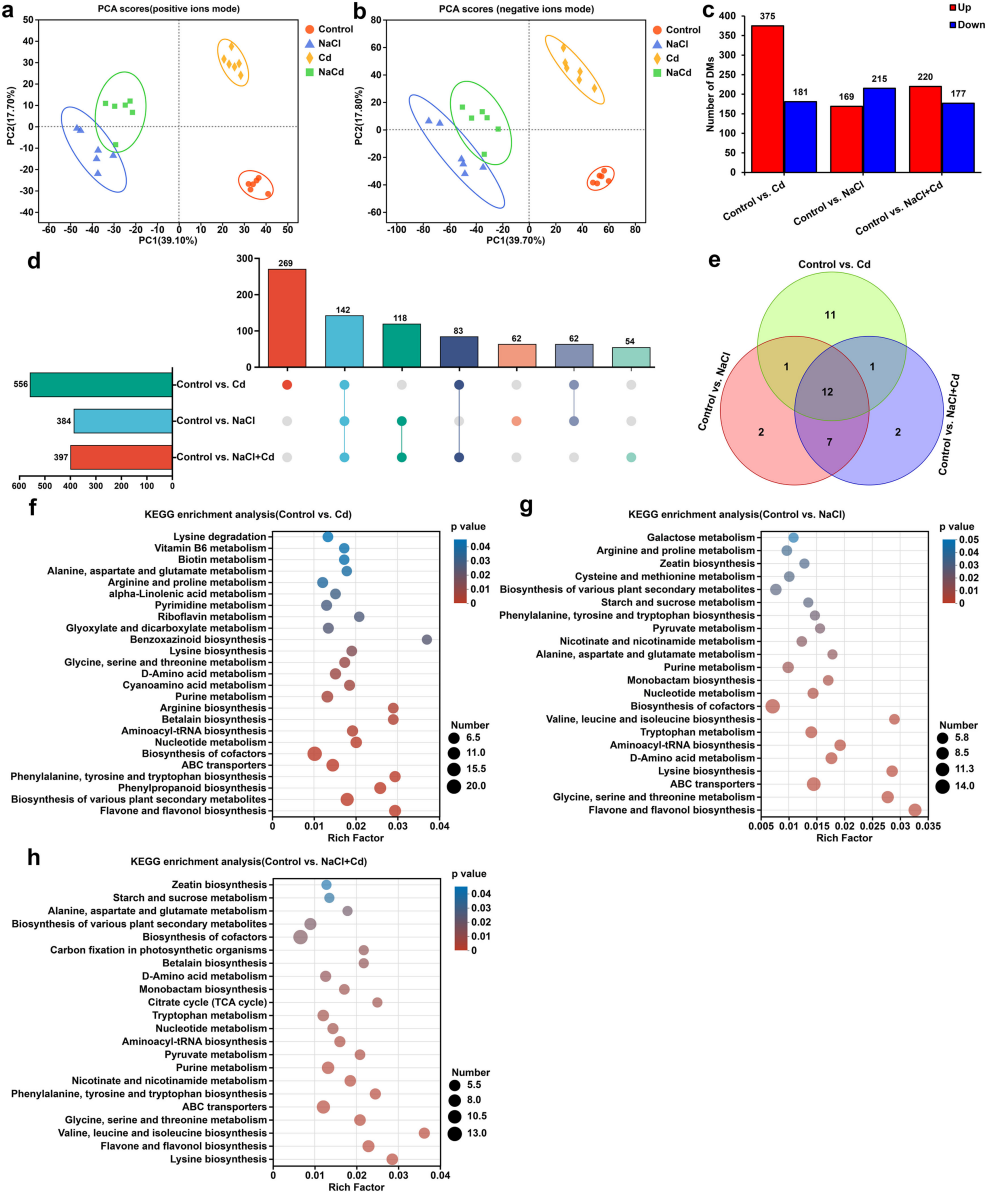
Metabolic profiling of wheat leaf subjected to Cd, NaCl, and NaCl+Cd stresses. **(A, B)** PCA score plot of metabolites obtained by positive and negative ion modes. **(C)** Statistical graph of DAMs under Cd, NaCl, and combined stresses compared with those in the control group. Red bars represent upregulated metabolites, and blue bars represent downregulated metabolites. **(D)** UpSet plot of DAMs. **(E)** Venn diagram of significantly enriched KEGG pathways among Cd, NaCl, and NaCl+Cd stressed groups. **(F-H)** KEGG enrichment analysis of DAMs in response to Cd **(F)**, NaCl **(G)**, and NaCl+Cd **(H)** stresses. The size of the dots represents the number of DAMs enriched in the pathway, and *p* < 0.05 represents statistical significance.

PCA revealed that the samples from the control, Cd, NaCl, and NaCl+Cd groups were separated, indicating that the metabolic profiles in the four groups were significantly different (**Figures 4A, B**). DAMs analysis based on VIP > 1.0 and *p* < 0.05 showed that 556 (375 increase and 181 decrease), 384 (220 increase and 177 decrease), and 397 (169 increase and 215 decrease) DAMs were identified under Cd, NaCl, and combined stresses, respectively, compared with those in the control group (**Figure 4C**). Among these DAMs, 142 were observed under all stress treatments, and 269, 62, and 54 were unique to Cd, NaCl and combined stresses, respectively (**Figure 4D**). KEGG pathway enrichment analysis of DAMs demonstrated that 25, 22, and 22 pathways were significantly enriched under Cd, NaCl, and combined stresses, respectively (**Figure 4E**). Among these, 12 pathways, including “Alanine, aspartate and glutamate metabolism”, “Phenylalanine, tyrosine and tryptophan biosynthesis”, “Lysine biosynthesis”, and “Glycine, serine and threonine metabolism”, were shared under all three stresses (**Figures 4F-H**). In addition to these common pathways, Cd stress specifically affected 11 other pathways, including “Phenylpropanoid biosynthesis”, “Riboflavin metabolism”, “Arginine biosynthesis”, and “Cyanoamino acid metabolism” (**Figure 4F**). NaCl stress specifically interfered with “Galactose metabolism” and “Cysteine and methionine metabolism” (**Figure 4G**). Unlike single stress, “Citrate cycle (TCA cycle)” and “Carbon fixation in photosynthetic organisms” were the unique pathways under combined stress (**Figure 4H**).

### 3.5 Effect of Cd, NaCl, and combined stresses on Chl content, Chl pathway, and photosynthetic proteins

**Figure 5:**
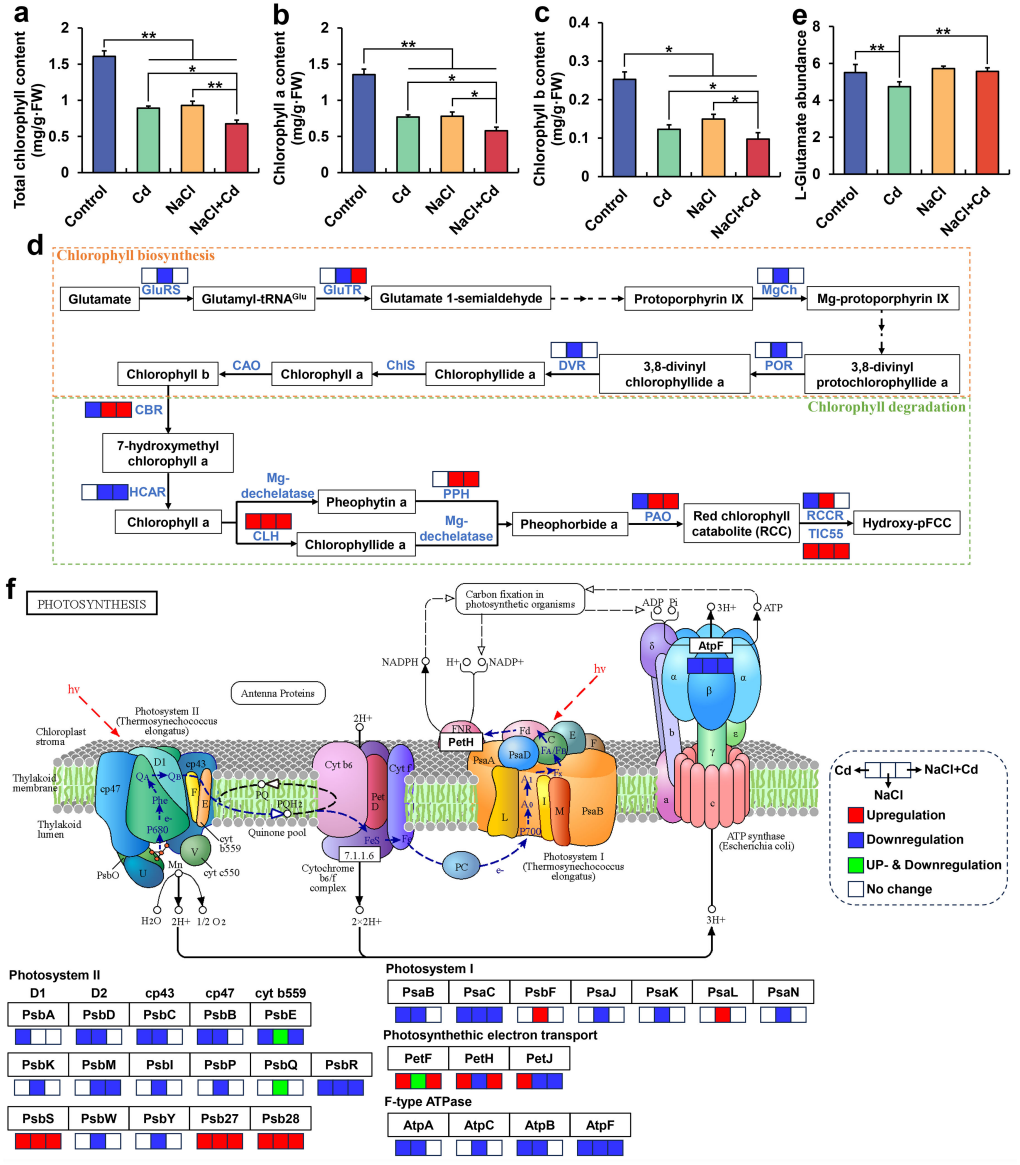
Chlorophyll content, chlorophyll-related gene expression, and photosynthesis-related gene expression in wheat seedlings under Cd, NaCl, and NaCl+Cd stresses. **(A)** Total Chl content. **(B)** Chl *a* content. **(C)** Ch *b* content. **(D)** Chl biosynthesis and degradation pathway. **(E)** Relative abundance of glutamate. **(F)** Photosynthesis pathway. Red represents upregulated DEGs, blue represents downregulated DEGs, and green represents up- and downregulated DEGs. Data are expressed as the means ± SD (n = 3 per group). * and ** represent *p* < 0.05 and *p* < 0.01, respectively.

The total Chl, Chl *a*, and Chl *b* contents in the leaves were significantly reduced under Cd, NaCl, and combined stresses, compared with those in the control group (**Figures 5A-C**). Notably, wheat plants under combined stress showed higher inhibition of these indicators than those under Cd or NaCl stress. Pearson’s correlation analysis showed that the total Chl, Chl *a*, and Chl *b* contents were positively correlated with PDW (**Supplementary Table S11**).

At the transcriptional level, NaCl stress significantly downregulated the expression of DEGs encoding glutamate-tRNA ligase (GluRS), glutamate-tRNA reductase (GluTR), Mg-chelatase (MgCh), NADPH-protochlorophyllide oxidoreductase (POR), and divinyl chlorophyllide a 8-vinyl-reductase (DVR) in the Chl biosynthesis pathway and upregulated the expression of DEGs encoding Chl b reductase (CBR), chlorophyllase (CLH), pheophytinase (PPH), pheophorbide a oxygenase (PAO), RCC reductase (RCCR), and TIC55 in the Chl degradation pathway (**Figure 5D**). NaCl+Cd stress significantly upregulated CBR, CLH, PPH, PAO, and TIC55 expressions in the Chl degradation pathway. Cd stress significantly upregulated the CLH expression but significantly downregulated the CBR, PAO, and RCCR expressions, and significantly reduced L-glutamate content at the metabolic level (**Figure 5E**). Moreover, 22 DEGs encoding 16 subunit proteins, 64 DEGs encoding 26 subunit proteins, and 22 DEGs encoding 11 subunit proteins related to the photosynthesis pathway were affected by Cd, NaCl, and combined stresses, respectively (**Figure 5F**). Specifically, 11 subunits related to photosystem II (PSII), PSI, and F-type ATPase were significantly downregulated under Cd stress. 21 subunits related to PSII, PSI, photosynthetic electron transport, and F-type ATPase were significantly downregulated under NaCl stress. 6 subunits related to PSII, PSI, photosynthetic electron transport, and F-type ATPase were significantly downregulated under combined stress.

### 3.6 Effect of Cd, NaCl and combined stresses on O_2_^•−^ and MDA contents

**Figure 6:**
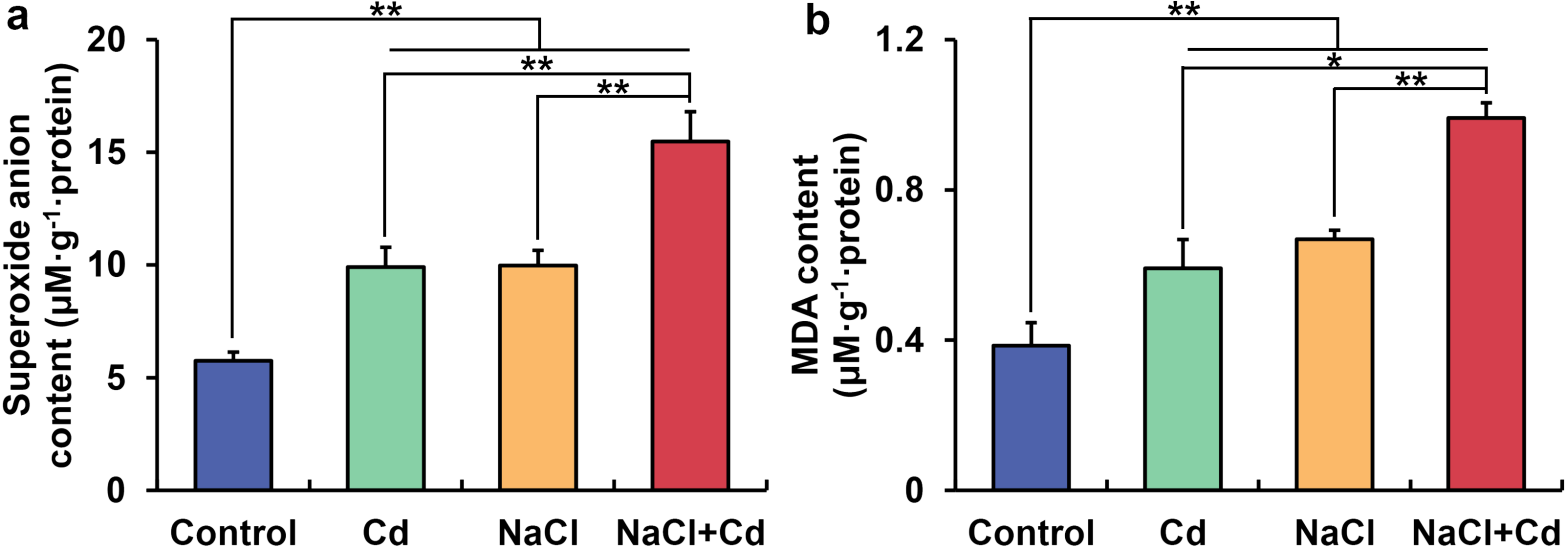
Contents of leaf superoxide anion **(A)** and malondialdehyde **(B)** in wheat seedlings under Cd, NaCl, and NaCl+Cd stresses. Data are expressed as the means ± SD (n=3 per group). * and ** represent *p* < 0.05 and *p* < 0.01, respectively.

O_2_^•−^ and MDA contents in the leaf were significantly increased under Cd, NaCl, and combined stresses compared with those in the control group (**Figures 6A, B**). Meanwhile, wheat plants under combined stress showed higher O_2_^•−^ and MDA contents than those under Cd or NaCl stress. Pearson’s correlation analysis showed that O^2•−^ and MDA contents were negatively correlated with PDW (**Supplementary Table S11**).

### 3.7 Integrative analysis of transcriptomics and metabolomics data

**Figure 7:**
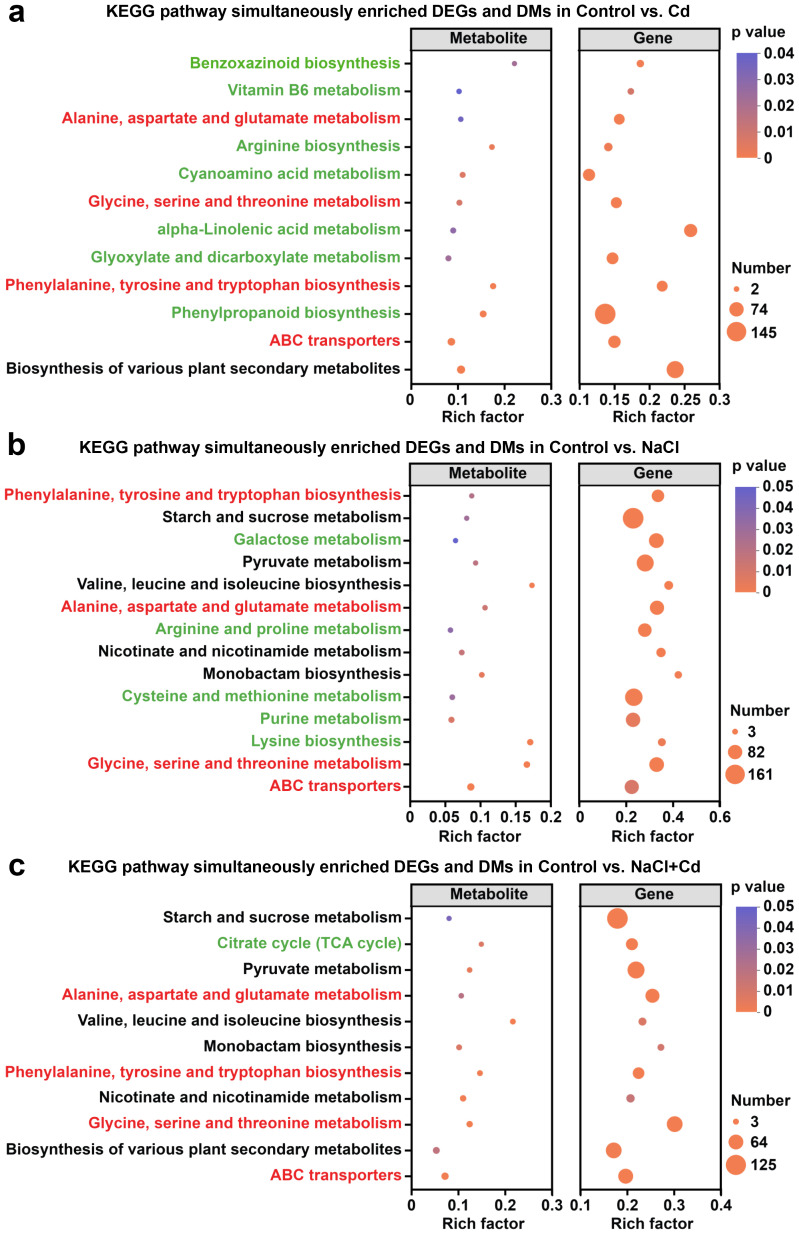
Integrative analysis of transcriptomics and metabolomics. **(A)** KEGG pathway simultaneously enriched DEGs and DAMs in Control *vs*. Cd. **(B)** KEGG pathway simultaneously enriched DEGs and DAMs in Control *vs*. NaCl. **(C)** KEGG pathway simultaneously enriched DEGs and DAMs in Control *vs*. NaCl+Cd. Red font represents common pathways, and green font represents unique pathways.

Integrated transcriptome and metabolome analyses were performed to identify the critical pathways in which the DEGs and DAMs participate. There were 12, 14, and 11 KEGG pathways that simultaneously enriched DEGs and DAMs in control *vs*. Cd, control *vs*. NaCl, and control *vs*. NaCl+Cd comparison groups, respectively (**Supplementary Figure S5**). In each comparison group, 4 common pathways were detected, with the “Phenylalanine, tyrosine and tryptophan biosynthesis” pathway exhibiting the most significant degree of enrichment (**Figure 7**). Furthermore, 7, 5, and 1 specific pathways were detected in control *vs*. Cd, control *vs*. NaCl, and control *vs*. NaCl+Cd comparisons, respectively (**Figure 7**). Based on the *p* < 0.01 and number of enriched DAMs and DEGs, “Phenylpropanoid biosynthesis” was the most representative unique pathway in the control *vs*. Cd comparison group (**Figure 7A**). “Purine metabolism” was the most representative unique pathway in the control *vs*. NaCl comparison group (**Figure 7B**). “TCA cycle” was the only unique pathway in the control *vs*. NaCl+Cd comparison group (**Figure 7C**). In addition, 5 common pathways were found between the NaCl stress and combined stress groups, indicating that the response of wheat to combined stress was highly similar to that under NaCl stress. Among these common pathways, “Starch and sucrose metabolism” was the most representative and enriched the most significant number of DAMs and DEGs (**Figures 7B, C**).

### 3.8 Expression patterns of DEGs and DAMs associated with phenylalanine, tyrosine and tryptophan biosynthesis and starch and sucrose metabolism

**Figure 8:**
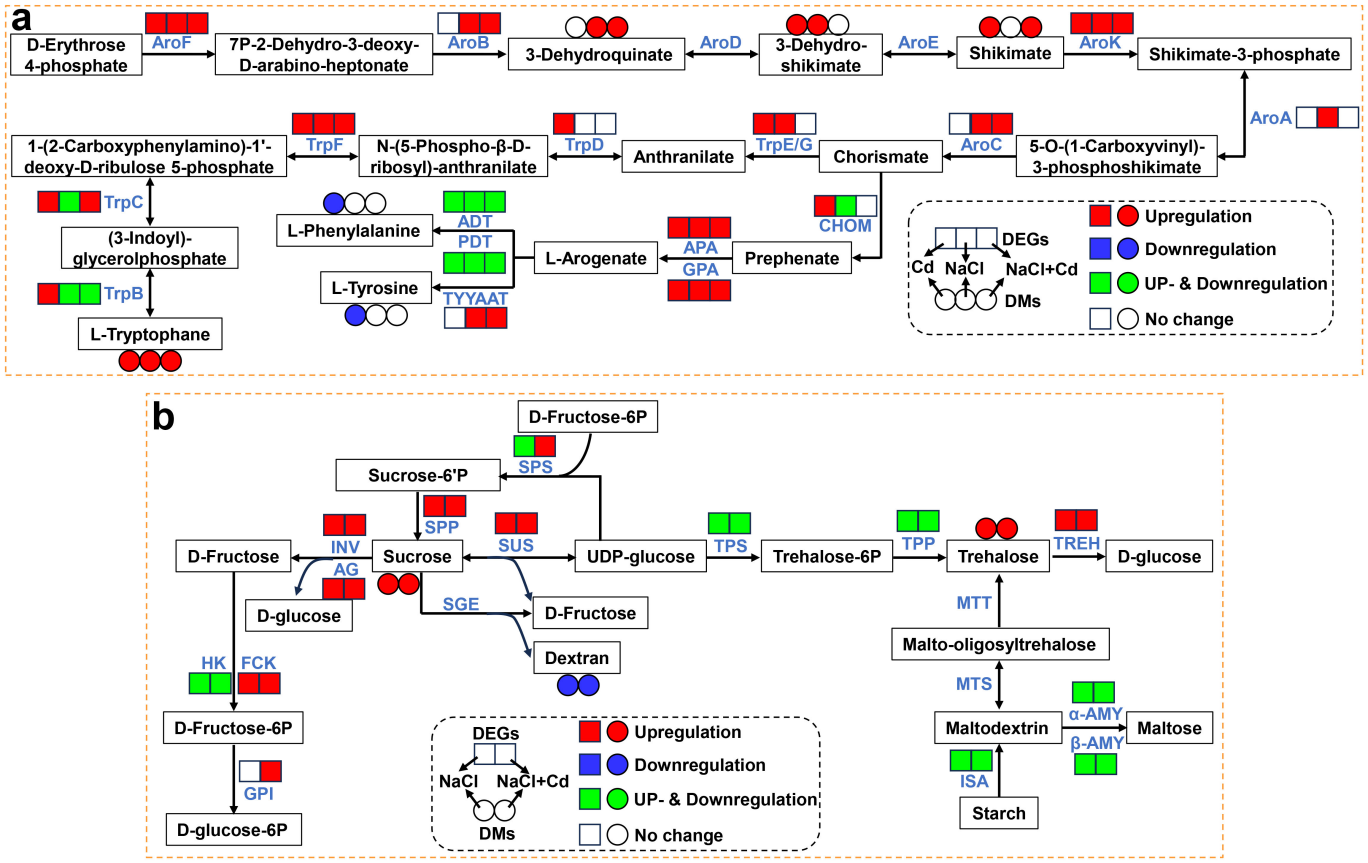
Effect of Cd, NaCl, and NaCl+Cd stresses on the genes and metabolites associated with phenylalanine, tyrosine and tryptophan biosynthesis, and starch and sucrose metabolism. **(A)** Phenylalanine, tyrosine and tryptophan biosynthesis pathway during Cd, NaCl, and NaCl+Cd stresses. **(B)** Starch and sucrose metabolism pathway during NaCl and NaCl+Cd stresses. Red represents upregulated DEGs or increased DAMs. Blue represents downregulated DEGs or decreased DAMs. Green represents up- and downregulated DEGs.

Under Cd, NaCl and combined stresses, many genes were significantly altered in the phenylalanine, tyrosine and tryptophan (Trp) biosynthesis pathway, especially those involved in Trp biosynthesis (**Figure 8A**; **Supplementary Table S12**). Specifically, the DEGs encoding AroF, AroK, and TrpF were significantly upregulated under all three stress treatments. Other DEGs encoding TrpB, TrpC, TrpD, TrpE/G, AroA, AroB, and AroC were significantly upregulated under one or two stress treatments. At the metabolic level, the Trp content increased significantly under all three stresses. The 3-dehydro-shikimate, shikimate, and 3-dehydroquinate contents were significantly increased under two stresses.

As shown in **Figure 8B** and **Supplementary Table S13**, the expressions of some major genes involved in starch and sucrose metabolism were significantly altered by NaCl and combined stresses. Notably, most DEGs showed consistent expression patterns under these two treatments, such as sucrose synthase (SUS), sucrose-6-phosphatase (SPP), trehalose-6-phosphate synthase (TPS), trehalose-6-phosphate phosphatase (TPP), and trehalose (TREH). At the metabolic level, sucrose and trehalose contents significantly increased, while dextran content significantly decreased.

### 3.9 Analysis of unique pathways under Cd, NaCl, and combined stresses

**Figure 9:**
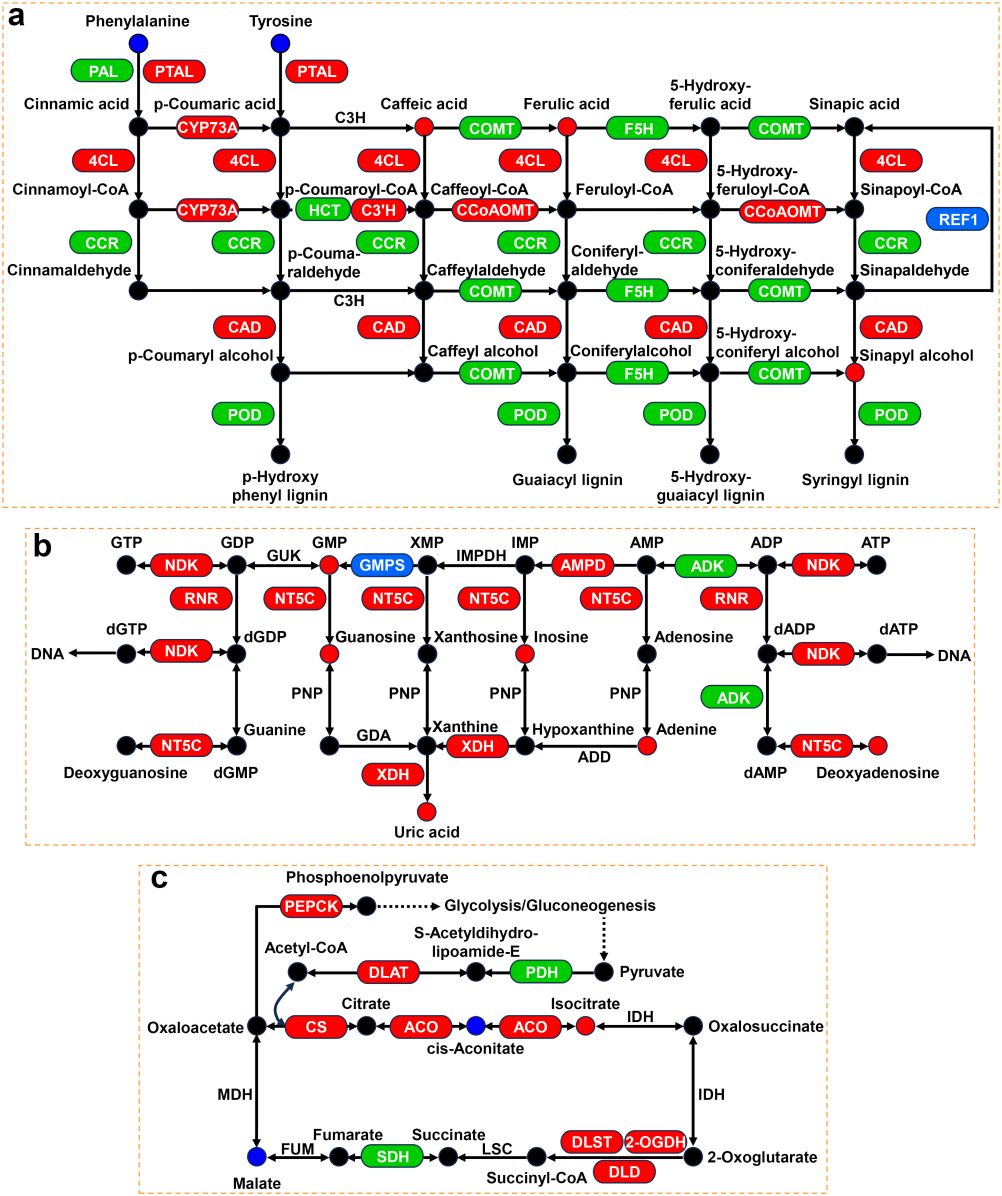
Expression profiles of genes and metabolites associated with phenylpropanoid biosynthesis, purine metabolism, and TCA cycle. **(A)** Phenylpropanoid biosynthesis pathway under Cd stress. **(B)** Purine metabolism pathway under NaCl stress. **(C)** TCA cycle pathway under NaCl+Cd stress. Red represents upregulated DEGs or increased DAMs. Blue represents downregulated DEGs or decreased DAMs. Green represents up- and downregulated DEGs.

In the phenylpropanoid biosynthesis pathway, 134 and 11 DEGs were significantly upregulated and downregulated, respectively, during Cd stress (**Figure 9A**; **Supplementary Table S14**). Specifically, the expression of genes encoding phenylalanine/tyrosine ammonia-lyase (PTAL), trans-cinnamate 4-monooxygenase (CYP73A), 4-coumarate-CoA ligase (4CL), 5-O-(4-coumaroyl)-D-quinate 3’-monooxygenase (C3’H), caffeoyl-CoA O-methyltransferase (CCoAOMT), and cinnamyl-alcohol dehydrogenase (CAD) was significantly upregulated. Meanwhile, the expression of genes encoding phenylalanine ammonia-lyase (PAL, 12 upregulated and 4 downregulated), caffeate O-methyltransferase (COMT, 6 upregulated and 1 downregulated), ferulate-5-hydroxylase (F5H, 7 upregulated and 1 downregulated), cinnamoyl-CoA reductase (CCR, 6 upregulated and 1 downregulated), shikimate O-hydroxycinnamoyltransferase (HCT, 1 upregulated and 1 downregulated), and peroxidase (POD, 76 upregulated and 2 downregulated) was also significantly affected. In addition, the levels of some pivotal metabolites, such as caffeic acid, ferulic acid, and sinapyl alcohol, were significantly increased.

Under NaCl stress, 74 DEGs and 6 DAMs were significantly altered in the purine metabolism pathway (**Figure 9B**; **Supplementary Table S14**). The levels of all DAMs were significantly increased, including GMP, guanosine, inosine, adenine, deoxyadenosine and uric acid. Furthermore, there was a significant upregulation of the expression of genes involved in these DAMs, such as nucleoside-diphosphate kinase (NDK), ribonucleoside-diphosphate reductase (RNR), 5’-nucleotidase (NT5C), AMP deaminase (AMPD), and xanthine dehydrogenase/oxidase (XDH).

Unlike under single stress treatment, combined stress explicitly affected the TCA cycle (**Figure 9C**; **Supplementary Table S14**). At the transcriptional level, the expression of genes encoding citrate synthase (CS), aconitate hydratase (ACO), 2-oxoglutarate dehydrogenase (2-OGDH), dihydrolipoyllysine-residue succinyltransferase (DLST), dihydrolipoyl dehydrogenase (DLD), and phosphoenolpyruvate carboxykinase (PEPCK), was significantly upregulated. Furthermore, the expression of genes encoding succinate dehydrogenase (SDH, 10 upregulated and 2 downregulated) and pyruvate dehydrogenase E1 component (PDH, 5 upregulated and 1 downregulated) was remarkably altered. At the metabolic level, isocitrate content was significantly increased, while cis-aconitate and malate contents were significantly decreased.

In the PPI network, COMT (TraesCS3B02G448600), CAD (TraesCS6D02G162800, TraesCS6B02G201600), and 4CL (TraesCS6B02G179900, TraesCS6A02G151700, TraesCS2D02G150400, TraesCS6D02G141700) in the phenylpropanoid biosynthesis pathway, AMPD (TraesCS2D02G084300, TraesCS2A02G087000, TraesCS2B02G101700) and ADK (TraesCS4B02G324400, TraesCS7A02G382500, TraesCS2B02G579200) in the purine metabolism pathway, and CS (TraesCS6B02G194700, TraesCS6D02G155800), SDH (TraesCS5A02G189800, TraesCS7A02G314000, TraesCS7D02G311200), and DLST (TraesCS2D02G039500) in the TCA cycle had the highest scores for betweenness centrality, thus reflecting their functional importance in the network (**Supplementary Figure S6**).

## 4 Discussion

### 4.1 Combined stress results in more severe growth inhibition and higher O2^•−^ and MDA levels than Cd or NaCl stress

Globally, soil salinity stress and Cd pollution have become non-negligible abiotic stresses. The retardation of crop growth due to Cd and salinity has been observed in wheat, maize, rice, and other crops (Huang et al., 2021; Sun et al., 2022; Zhang et al., 2023). Consistent with the results, in the present study, we found significant decreases in PRL, SH, and PDW in Cd- and NaCl-stressed wheat seedlings. Wheat plants under combined stress exhibited lower PRL, SH and PDW than those under single stress, indicating that combined stress caused more severe damage to wheat growth. Similar results have been observed in other wheat cultivars, quinoa, and *Spartina alterniflora* exposed to combined Cd and salinity stress (Abdal et al., 2023; Chai et al., 2013; Shafi et al., 2009). Generally, abiotic stress causes the accumulation of large amounts of reactive oxygen species (ROS) in plants, resulting in severe oxidative damage to proteins, DNA, and lipids (Das and Roychoudhury, 2014). Our study observed significant increases in O_2_^•−^ and MDA contents under all stress treatments, with combined stress having the highest contents. O_2_^•−^ is usually the first formed ROS, and can be converted into more reactive and toxic OH• and ^1^O2, leading to membrane lipid peroxidation (Halliwell, 2006). Therefore, these results indicated that combined stress had more serious oxidative damage than those of NaCl or Cd stress, which may be one of the reasons for the more severe growth inhibition. The positive correlation among PDW, O_2_^•−^, and MDA further confirmed this.

### 4.2 Cd increases Na^+^ content, whereas NaCl reduces Cd^2+^ content

Salinity stress usually results in Na^+^ accumulation in plants. In this study, high concentrations of Na^+^ were observed in NaCl-treated wheat plants. Plants have evolved a series of salinity-resistance strategies to adapt to salinity stress. Among these, the SOS pathway is one of the most important, and involves three proteins: plasma membrane Na^+^/H^+^ antiporter SOS1, calcineurin B-like (CBL)-interacting protein kinase SOS2, and calcium-binding protein SOS3 (Zhu, 2000). When plants sense excess Na^+^, SOS3 activates and recruits SOS2 to the plasma membrane. SOS2 phosphorylates and activates SOS1 to exclude excessive Na^+^ from the cytosol (Ali et al., 2023; Ji et al., 2013). In this study, the SOS1, SOS2, and SOS3 transcriptional levels were significantly upregulated under NaCl and combined stresses, indicating that wheat plants activated the SOS pathway under these two stresses. Notably, the SOS1 upregulation under the combined stress was only 0.74-fold that under salinity stress alone, indicating that Cd may weaken the SOS1 expression, thereby inhibiting Na^+^ efflux and increasing Na^+^ content under combined stress. In addition to SOS-mediated Na^+^ efflux, plants transport Na^+^ into the vacuoles for sequestration by the vacuole-localized NHXs under salt stress (Xiao and Zhou, 2023). In this study, some DEGs encoding vacuolar NHXs were significantly upregulated under NaCl and combined stresses, indicating that wheat plants initiated vacuolar compartmentalization of Na^+^.

In contrast to the Na^+^ content, the Cd^2+^ content was significantly lower under combined stress than that under Cd stress. Plants contain many proteins involved in Cd uptake and transport. Among them, Nramp and ZIP are responsible for Cd uptake and translocation to aboveground parts (Sasaki et al., 2012; Hou et al., 2023). In this study, 5 DEGs encoding Nramp and 8 DEGs encoding ZIPs were significantly upregulated under Cd stress, whereas 2 DEGs encoding Nramp and most DEGs encoding ZIP were significantly downregulated under combined stress. Therefore, the low Cd accumulation under combined stress may be attributed to the partial downregulation of Nramp and ZIP gene expression by NaCl.

### 4.3 Cd, NaCl, and combined stresses inhibit photosynthetic pigments and photosynthetic proteins

Chl is essential for photosynthesis in most plants, where Chl *a* converts light energy into chemical energy, and Chl *b* captures and transmits light energy (Trukhachev et al., 2022). In this study, Cd, NaCl, and combined stresses sharply suppressed the total Chl, Chl *a*, and Chl *b* contents. In plants, Chl biosynthesis commences with the precursor 5-aminolevulinic acid (ALA), which is generated from glutamate under catalysis by GluRS, GluTR and glutamate-1-semialdehyde transaminase (Tanaka et al., 2011). ALA is then gradually converted into protoporphyrin IX (Proto IX), which is further catalyzed by the enzymes MgCh, POR, DVR, Chl synthetase (ChlS), and chlorophyllide an oxygenase (CAO) to produce Chl *a* and Chl *b* (Masuda and Takamiya, 2004; Liu and Zheng, 2008). In this study, we observed that many DEGs involved in the Chl biosynthesis pathway under NaCl stress were significantly downregulated, including GluRS, GluTR, MgCh, POR, and DVR, indicating that NaCl stress inhibited Chl biosynthesis. Contrastingly, many DEGs involved in Chl degradation, such as CBR, CLH, PPH, PAO, and TIC55, were significantly upregulated under NaCl and NaCl+Cd stresses. In the Chl degradation pathway, Chl *b* is first converted to Chl *a* through a two-step reaction catalyzed by CBR and hydroxymethyl chlorophyll a reductase (HCAR) (Horie et al., 2009; Meguro et al., 2011). Chl *a* is converted into Pheide a by removing phytol and Mg^2+^ catalyzed by Mg-dechelatase (MDCase) and PPH or CLH enzymes (Lin and Charng, 2021). Pheide a undergoes ring opening catalyzed by PAO to generate linear red chlorophyll metabolites (RCC), which are further converted into colorless pFCC and hpFCC under the catalysis of RCC reductase (RCCR) and TIC55 (Bhattacharyya et al., 2015; Kuai et al., 2018). Therefore, we suggested that NaCl stress reduced the Chl content by inhibiting Chl biosynthesis and activating Chl degradation, whereas NaCl+Cd reduced the Chl content by activating Chl degradation. Unlike NaCl and NaCl+Cd stresses, Cd stress significantly downregulated the expression of some DEGs related to Chl degradation, such as CBR, PAO, and RCCR. Notably, we found that the L-glutamate content, the initial substrate for Chl biosynthesis, was significantly decreased under Cd stress, which may partially explain why Cd stress reduced Chl content.

Photosynthesis plays a decisive role in plant growth and development, mainly involving four pigment-protein complexes: photosystem I (PSI), photosystem II (PSII), cytochrome b6f complex, and ATP synthase complex (Allen et al., 2011). PSI and PSII are multi-subunit membrane protein complexes, where PSII catalyzes water splitting, oxygen evolution, and plastoquinone reduction, whereas PSI generates reducing power for the reduction of NADP^+^ to NADPH (Gao et al., 2018). ATP synthase participates in ATP synthesis, mediated by transmembrane proton motive force (Zhang et al., 2015). In this study, multiple genes encoding PSII, PSI, and F-type ATPase were significantly downregulated under Cd, NaCl, and combined stresses, indicating the inhibition of photosynthetic proteins, which may further lead to a decline in photosynthesis. In summary, the stress-induced inhibition of photosynthetic pigments and proteins in this study may affect photosynthesis and carbon fixation, ultimately resulting in growth inhibition in wheat seedlings.

### 4.4 Cd, NaCl, and combined stresses activate Trp biosynthesis and increase Trp content

Under all three stresses, there was a significant upregulation of DEGs related to Trp biosynthesis, indicating that Cd, NaCl, and combined stresses activated Trp biosynthesis. The observed increase in Trp content under all three stresses further supported our hypothesis. Trp is an essential amino acid for protein synthesis in living organisms and has recently been shown to play an important role in protecting plants against abiotic stress (Godoy et al., 2021; Zemanová et al., 2014). For instance, exogenous Trp improved Cd tolerance in broccoli (*Brassica oleracea* var. italica) and Arabidopsis (*Arabidopsis thaliana*) (Jiang et al., 2022; Li and Qi, 2023). The application of Trp alleviated the detrimental effects of salt stress in red pepper (*Capsicum annuum* L.) and sugar beet (*Beta vulgaris* L.) (Hozayn et al., 2020; Jamil et al., 2018). Therefore, activating Trp biosynthesis may improve wheat adaptation to Cd, NaCl, and combined stresses.

### 4.5 Starch and sucrose metabolism plays an important role under NaCl and combined stresses

In this study, numerous DEGs involved in starch and sucrose metabolism were significantly altered, resulting in increased sucrose and trehalose contents under NaCl and combined stresses. Similarly, increased levels of soluble sugars (sucrose, trehalose, glucose, and fructose) have been observed in many plants under salinity, drought, water, chilling, and nutrient deficiency stresses (Martínez-Noël and Tognetti, 2018; Saddhe et al., 2021). Accumulated soluble sugars act as osmo-protectants in maintaining osmotic homeostasis, stabilizing membrane structure, and scavenging toxic ROS against different stresses (Amist and Singh, 2020; Singh et al., 2015). Exogenous application of sucrose and trehalose improved plant tolerance to salinity and heat stresses (Gong and Chen, 2021; Yang et al., 2014). Therefore, the accumulation of sucrose and trehalose observed in this study may help wheat better cope with salinity and combined stresses.

### 4.6 Phenylpropanoid biosynthesis plays a unique role under Cd stress

For phenylpropanoid biosynthesis, multitudinous DEGs encoding PTAL, CYP73A, 4CL, C3’H, CCoAOMT, CAD, COMT, F5H, CCR, and POD were significantly upregulated under Cd stress, indicating that Cd stress activated the pathway. As one of the most important metabolic pathways in plants, the phenylpropanoid biosynthesis pathway produces many secondary metabolites, including phenolic acids possessing redox properties (Zhang and Liu, 2015; Sharma et al., 2019). In this study, there were significant increases in caffeic acid (CA) and fumaric acid (FA) under Cd stress. CA is synthesized from phenylalanine or tyrosine under the sequential catalysis of PAL/PTAL, CYP73A, and C3’H, and then transformed into FA via COMT catalysis (Riaz et al., 2019). They are well-known as potential antioxidants that improve abiotic stress resistance in plants by scavenging ROS (Chen et al., 2020; Shu et al., 2022; Wan et al., 2015). Therefore, wheat may increase CA and FA levels to cope with Cd-induced oxidative damage by activating the phenylpropanoid biosynthesis pathway.

### 4.7 Purine metabolism plays a unique role under NaCl stress

Under NaCl stress, all DAMs, including uric acid, and nearly all DEGs in the purine metabolism pathway were markedly increased or upregulated in the present study, suggesting that the purine metabolism pathway plays a positive role in the response to NaCl stress. Our results were consistent with those of previous studies showing that the activation of enzymes and increased levels in intermediary metabolites are associated with purine metabolism in stressed plants (Alamillo et al., 2010; Liu et al., 2020; Rose et al., 2012; Sagi et al., 1998). Purine metabolism not only recycles nitrogen by disintegrating the purine ring, but also produces uric acid which functions to scavenge ROS (Ma et al., 2016; Soltabayeva et al., 2018; Werner and Witte, 2011). Sun et al. (2021) reported that exogenous application of uric acid elevated the resistance of apple plants to salinity stress by enhancing ROS scavenging. Therefore, we suggested that wheat plants may increase intermediate metabolites, including uric acid, by activating the purine metabolism pathway to protect plants from oxidative damage due to NaCl stress.

### 4.8 TCA cycle plays a unique role under combined stress

Under combined stress, the “TCA cycle” was the only specific pathway observed. As the epicenter of cell metabolism, the TCA cycle is essential in energy and material metabolism (Zhang and Fernie, 2023). In this study, critical genes related to the TCA cycle, such as CS, ACO, 2-OGDH, DLST, DLD, and SDH, were consistently upregulated when subjected to combined stress. CS initiates the first step of the TCA cycle and catalyzes the condensation of acetyl-CoA and oxaloacetate to form citrate, which is then converted to isocitrate under the action of ACO (Lehmann et al., 2009; Schmidtmann et al., 2014). 2-OGDH, DLST, and DLD are components of the 2-OGDH complex, and convert 2-oxoglutarate into succinyl CoA and generate further CO_2_ and NADH (Araújo et al., 2012). SDH participates in the oxidation of succinate to fumarate with the simultaneous reduction of flavin adenine dinucleotide (FAD) to FADH2 (Huang and Millar, 2013). NADH and FADH2 molecules pass their electrons into the electron transport chain and undergo oxidative phosphorylation to generate most of the energy (ATP) required for plant growth, cell division, and other physiological processes (Ghifari et al., 2023). Therefore, the upregulation of genes involved in the TCA cycle may produce more energy provision for wheat plants to counteract the combined stress.

## 5 Conclusions

Our study showed that combined stress caused more severe adverse effects on wheat growth and Chl content and produced more O_2_^•−^ and MDA contents than those under NaCl or Cd stress. Stress-induced decrease in Chl levels may be related to the inhibition of Chl biosynthesis, activation of Chl degradation, or a decline in glutamate content. Integrated transcriptomics and metabolomics analyses revealed similarities and differences in the responses of wheat to single and combined stresses. All three stresses activated Trp biosynthesis, increasing Trp levels. Meanwhile, Cd stress specifically activated phenylpropanoid biosynthesis and increased CA and FA contents. NaCl stress specifically activated purine metabolism and enhanced intermediate metabolite levels. Combined stress explicitly activated the TCA cycle, leading to increased energy provision. In addition, NaCl and combined stresses affected starch and sucrose metabolism, resulting in the accumulation of sucrose and trehalose.

## Data Availability

The datasets presented in this study can be found in online repositories. The names of the repository/repositories and accession number(s) can be found in the article/**Supplementary Material**.
