# Supplementary material for: Integrated transcriptomics, metabolomics and physiological analyses reveal differential response mechanisms of wheat to cadmium and/or salinity stress
Source: Front Plant Sci. 2024 Oct 1;15:1378226. doi: 10.3389/fpls.2024.1378226 (PMC11473431; doi:10.3389/fpls.2024.1378226)
Supplement: Supplementary file 1 [file DataSheet1.pdf]

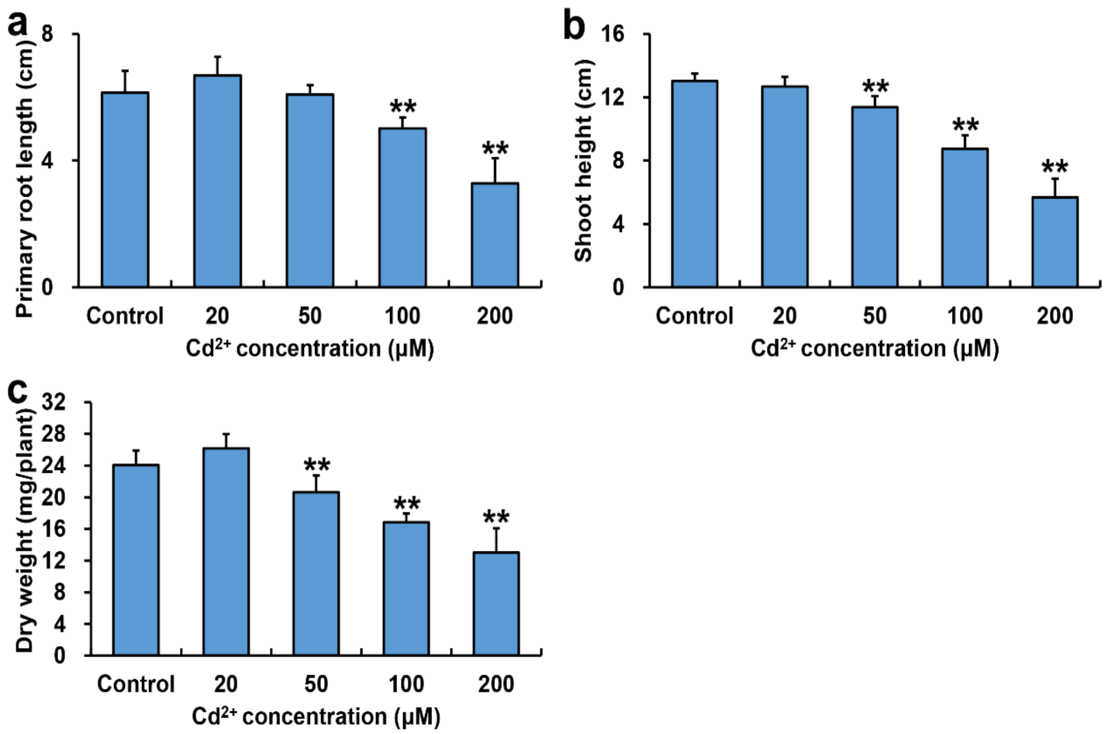

FIGURE S1 Effects on the growth of wheat exposed to the different concentrations of Cd^{2+} for 7 days.

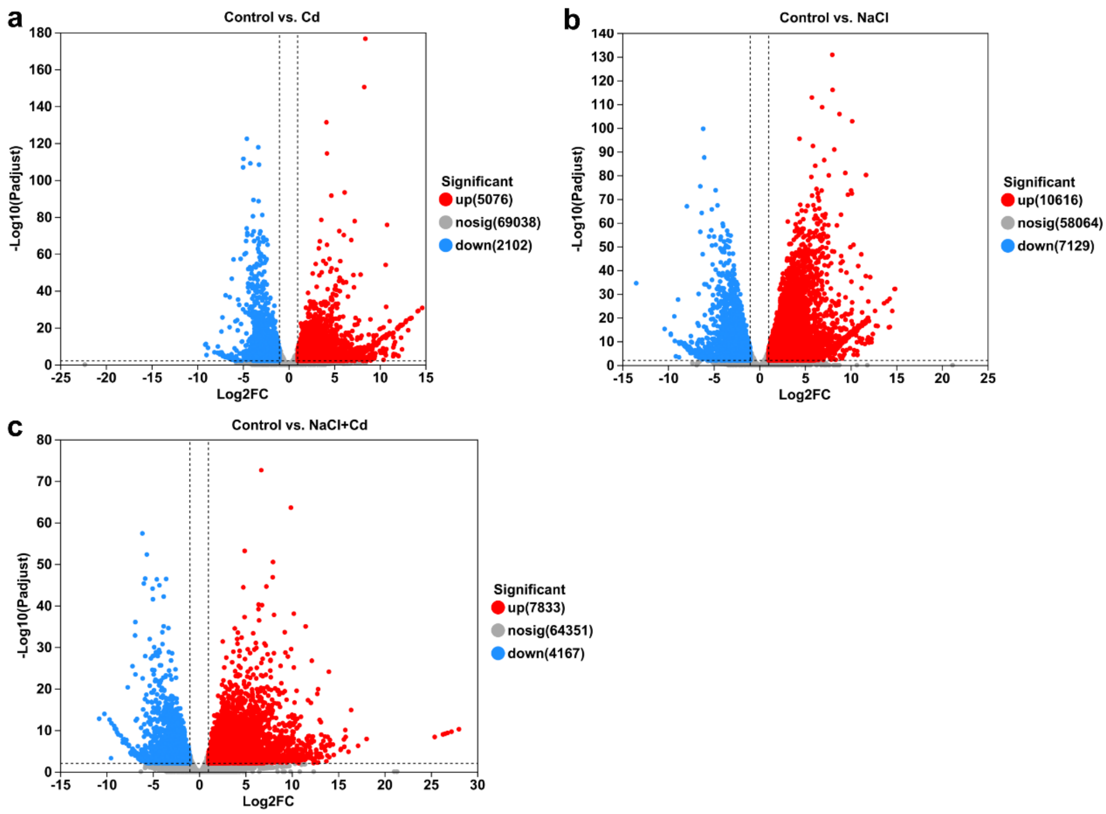

Fig.S2 Volcano plot of differentially expressed genes (DEGs) in Control vs. Cd (a), Control vs. NaCl (b), and Control vs. NaCl+Cd (c).

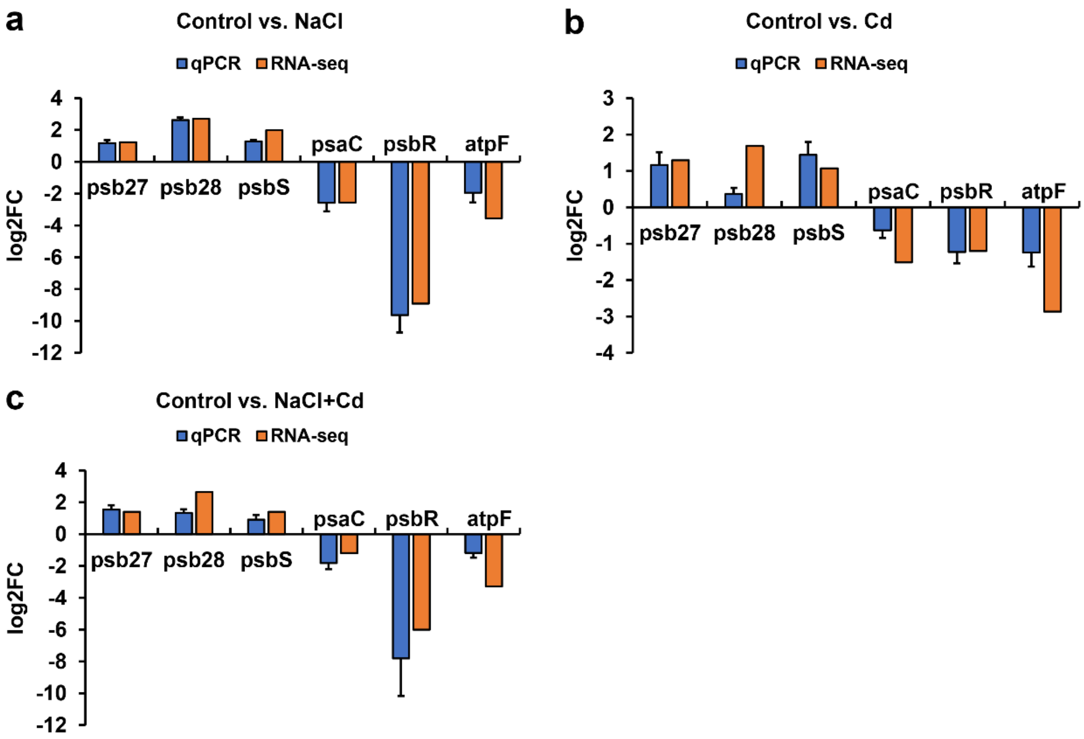

Fig.S3 Validation of RNA-seq data by qPCR.

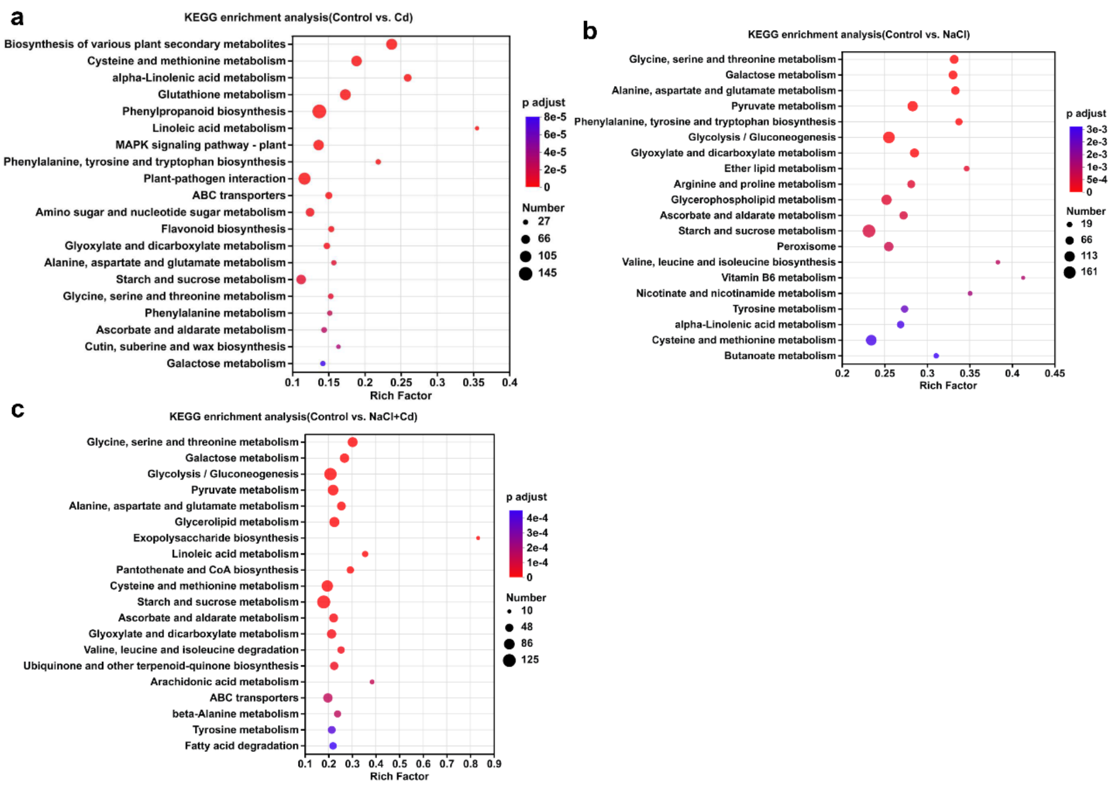

Fig.S4 KEGG enrichment analysis of DEGs in response to Cd (a), NaCl (b) and combined (c) stresses. The size of the dots represents the number of DEGs enriched in the pathway, and *p* adjust < 0.05 represents statistical significance.

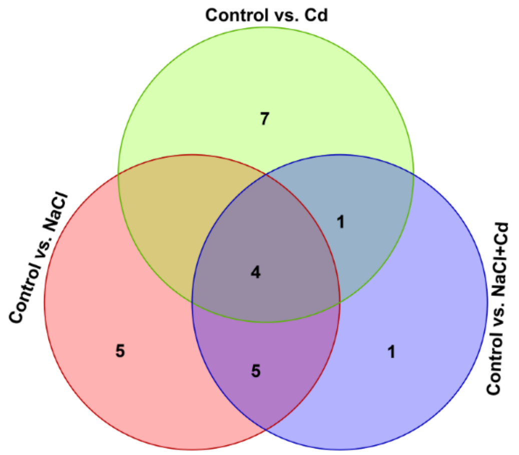

Fig.S5 Venn diagram of KEGG pathways that simultaneously enriches DEGs and DMs between Control vs. Cd, Control vs. NaCl, and Control vs. NaCl+Cd.
