# Supplementary material for: Integrated transcriptomics, metabolomics and physiological analyses reveal differential response mechanisms of wheat to cadmium and/or salinity stress
Source: Front Plant Sci. 2024 Oct 1;15:1378226. doi: 10.3389/fpls.2024.1378226 (PMC11473431; doi:10.3389/fpls.2024.1378226)
Supplement: Supplementary file 2 [file DataSheet2.pdf]

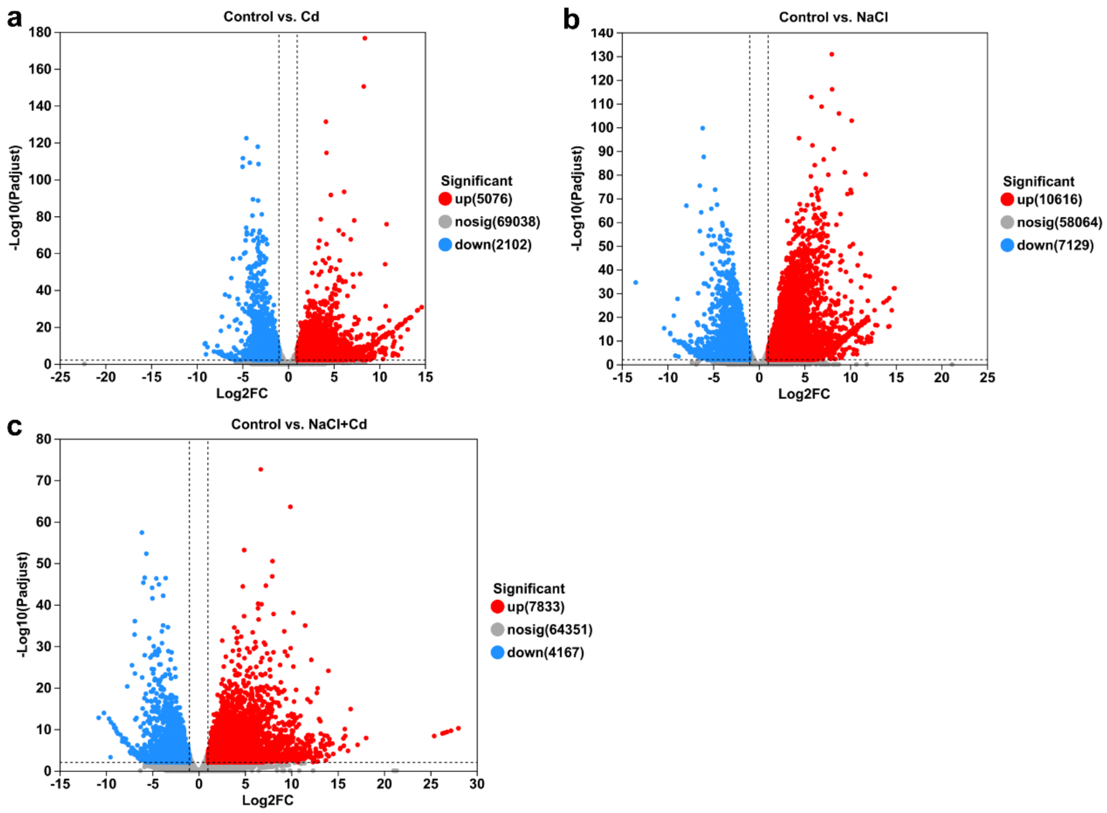

FIGRUE S2 Volcano plot of differentially expressed genes (DEGs) in Control vs. Cd **(a)**, Control vs. NaCl **(b)**, and Control vs. NaCl+Cd **(c)**.
