# Supplementary figures and images for: Integrated transcriptomics, metabolomics and physiological analyses reveal differential response mechanisms of wheat to cadmium and/or salinity stress
Source: Front Plant Sci. 2024 Oct 1;15:1378226. doi: 10.3389/fpls.2024.1378226 (PMC11473431; doi:10.3389/fpls.2024.1378226)

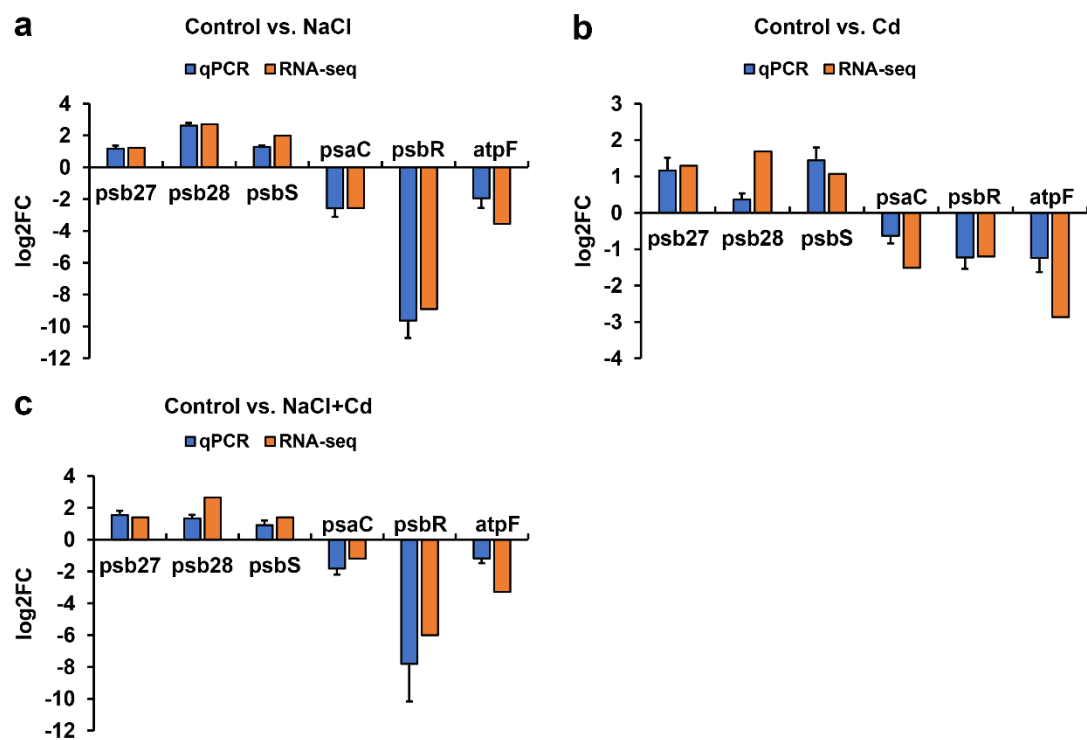

FIGURE S3 Validation of RNA-seq data by qPCR.

Supplement: Supplementary file 3 [file DataSheet3.pdf]
