# Supplementary material for: Integrated transcriptomics, metabolomics and physiological analyses reveal differential response mechanisms of wheat to cadmium and/or salinity stress
Source: Front Plant Sci. 2024 Oct 1;15:1378226. doi: 10.3389/fpls.2024.1378226 (PMC11473431; doi:10.3389/fpls.2024.1378226)
Supplement: Supplementary file 4 [file DataSheet4.pdf]

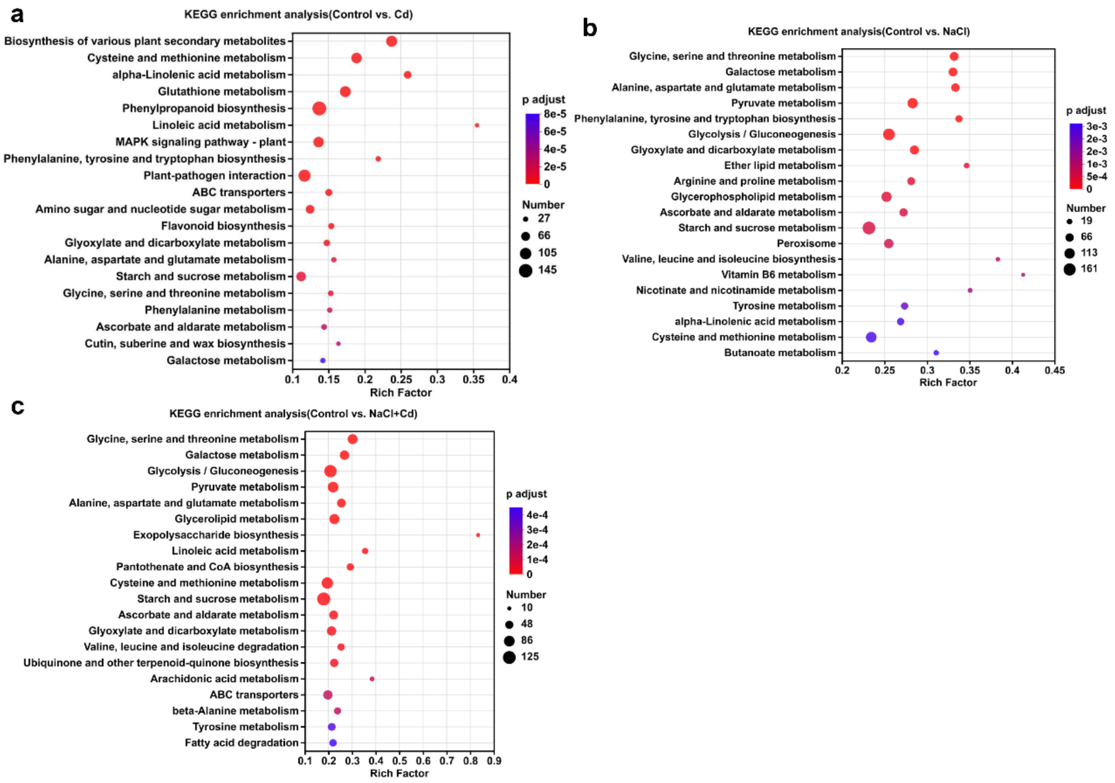

FIGURE S4 KEGG enrichment analysis of DEGs in response to Cd **(a)**, NaCl **(b)** and combined **(c)** stresses. The size of the dots represents the number of DEGs enriched in the pathway, and *p* adjust < 0.05 represents statistical significance.
