# Supplementary material for: Integrated transcriptomics, metabolomics and physiological analyses reveal differential response mechanisms of wheat to cadmium and/or salinity stress
Source: Front Plant Sci. 2024 Oct 1;15:1378226. doi: 10.3389/fpls.2024.1378226 (PMC11473431; doi:10.3389/fpls.2024.1378226)
Supplement: Supplementary file 5 [file DataSheet5.pdf]

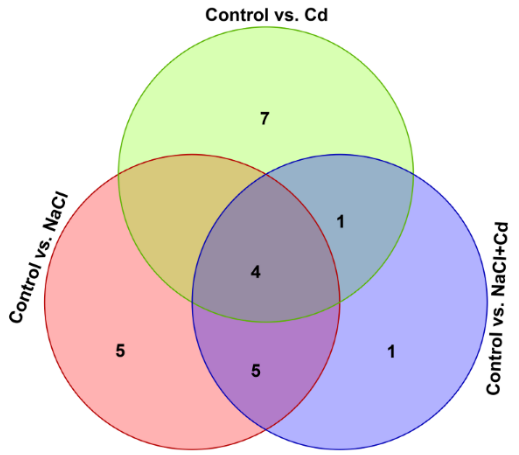

FIGURE S5 Venn diagram of KEGG pathways that simultaneously enriches DEGs and DMs between Control vs. Cd, Control vs. NaCl, and Control vs. NaCl+Cd.
