# Supplementary material for: Integrated transcriptomics, metabolomics and physiological analyses reveal differential response mechanisms of wheat to cadmium and/or salinity stress
Source: Front Plant Sci. 2024 Oct 1;15:1378226. doi: 10.3389/fpls.2024.1378226 (PMC11473431; doi:10.3389/fpls.2024.1378226)
Supplement: Supplementary file 6 [file DataSheet6.pdf]

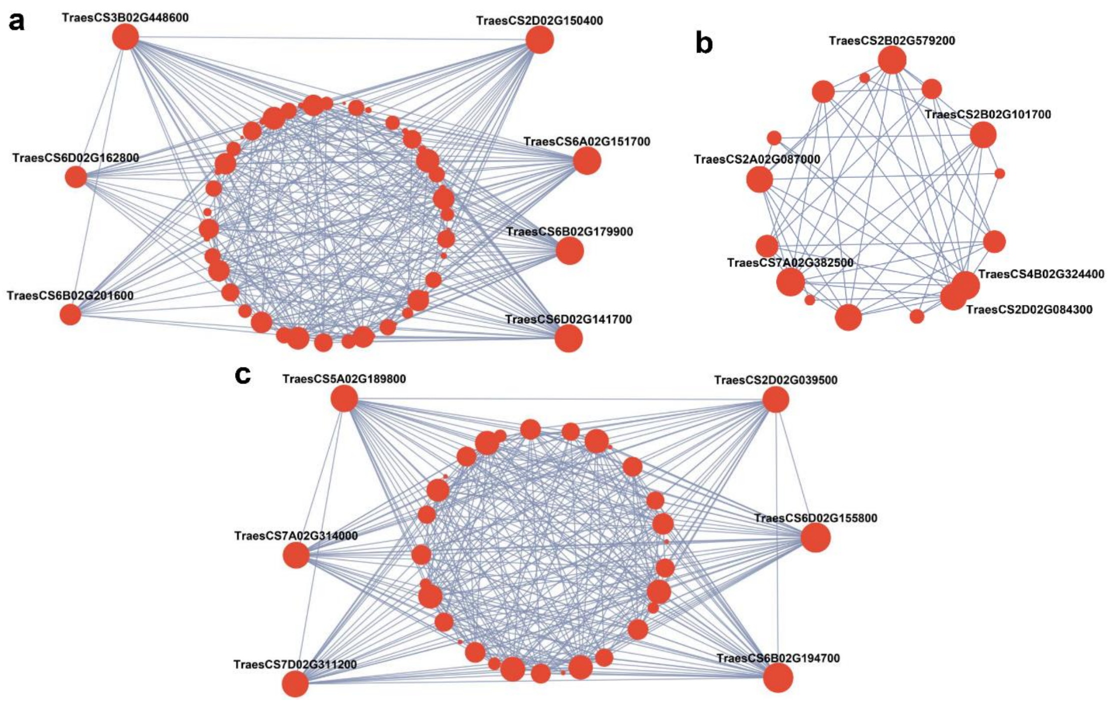

FIGURE S6 The PPI network analysis of DEGs involved in phenylpropanoid biosynthesis (a), purine metabolism (b), and TCA cycle (c) under Cd, NaCl and NaCl+Cd stresses.
