# Supplementary material for: Integrated transcriptomics, metabolomics and physiological analyses reveal differential response mechanisms of wheat to cadmium and/or salinity stress
Source: Front Plant Sci. 2024 Oct 1;15:1378226. doi: 10.3389/fpls.2024.1378226 (PMC11473431; doi:10.3389/fpls.2024.1378226)
Supplement: Supplementary file 7 [file DataSheet7.pdf]

Supplementary Methods

Extraction of chlorophyll

Briefly, 0.1 g fresh leaf samples were submerged in 8 mL dimethyl sulfoxide (DMSO) in 15 mL centrifuge tubes. After sealing, these tubes were transferred to an incubator and incubated in the dark for 1 h at 65 °C. The extract solution was then replenished with DMSO to a total volume of 10 mL.

RNA-seq library construction

Messenger RNA from total RNA was isolated using oligo (dT) beads and fragmented using fragmentation buffer. Then double-stranded cDNA was synthesized with random hexamer primers (Illumina, San Diego, USA) using a SuperScript double-stranded cDNA synthesis kit (Invitrogen, CA, USA). The produced cDNA was end-repaired, phosphorylated and added ‘A’ base according to Illumina’s library construction protocol. Libraries were size-selected for cDNA target fragments (300 bp) on 2% Low Range Ultra Agarose, followed by 15 cycles of PCR amplification using Phusion DNA polymerase (NEB, Ipswich, USA).

UPLC-MS/MS analysis

UPLC-MS/MS analysis was performed on an Vanquish Horizon UPLC system coupled with a Q Exactive HF-X mass spectrometer (Thermo Fisher). Specifically, the Vanquish Horizon UPLC system was equipped with an ACQUITY HSS T3 column (100 mm × 2.1 mm i.d., 1.8 μm; Waters, Milford, USA). The mobile phases consisted of 0.1% formic acid in water/acetonitrile (95/5, v/v) (A) and 0.1% formic acid in acetonitrile/isopropanol/water (47.5/47.5/5, v/v/v) (B). The column oven temperature

was set to 40 °C. The injection volume was 3 μL. The gradient elution conditions of mobile phases were summarized in Supplementary Table S2. The mass spectrometric data were collected using a Thermo UPLC-Q Exactive HF-X mass spectrometer with an electrospray ionization (ESI) source operating in positive and negative modes. Detailed MS parameters are shown in Supplementary Table S3.
