# Supplementary material for: Integrated transcriptomics, metabolomics and physiological analyses reveal differential response mechanisms of wheat to cadmium and/or salinity stress
Source: Front Plant Sci. 2024 Oct 1;15:1378226. doi: 10.3389/fpls.2024.1378226 (PMC11473431; doi:10.3389/fpls.2024.1378226)
Supplement: Supplementary file 8 [file DataSheet8.pdf]

Supplementary Results

As shown in Figure S4, there was a substantial overlap (7 pathways) between the three stresses, such as “starch and sucrose metabolism”, “glycine, serine and threonine metabolism”, and “alanine, aspartate and glutamate metabolism”. Additionally, 9 pathways were specifically enriched under Cd stress, such as “glutathione metabolism”, “phenylpropanoid biosynthesis”, and “MAPK signaling pathway – plant” (Figure S4a). 8 pathways were specifically enriched under NaCl stress, such as “arginine and proline metabolism”, “glycerophospholipid metabolism”, and “valine, leucine and isoleucine biosynthesis” (Figure S4b). 8 pathways were specifically enriched under combined stress, such as “glycerolipid metabolism”, “valine, leucine and isoleucine degradation”, and “arachidonic acid metabolism” (Figure S4c).
